# SONG: A Multi-Objective Evolutionary Algorithm for Delay and Energy Aware Facility Location in Vehicular Fog Networks

**DOI:** 10.3390/s23020667

**Published:** 2023-01-06

**Authors:** Md. Muzakkir Hussain, Ahmad Taher Azar, Rafeeq Ahmed, Syed Umar Amin, Basit Qureshi, V. Dinesh Reddy, Irfan Alam, Zafar Iqbal Khan

**Affiliations:** 1 Department of Computer Science and Engineering, SRM University, Amaravati 522502, India; 2 College of Computer and Information Sciences, Prince Sultan University, Riyadh 11586, Saudi Arabia; 3 Faculty of Computers and Artificial Intelligence, Benha University, Benha 13511, Egypt; 4 Department of CSE, Koneru Lakshmaiah Education Foundation, Vaddeswaram 522302, India; 5 Department of Computer Science and Engineering, Delhi Technological University, Delhi 110042, India

**Keywords:** intelligent transportation systems, vehicular fog computing, Genetic Algorithm, vehicular ad hoc network, SDG7, SDG9, SDG11, SDG12

## Abstract

With the emergence of delay- and energy-critical vehicular applications, forwarding sense-actuate data from vehicles to the cloud became practically infeasible. Therefore, a new computational model called Vehicular Fog Computing (VFC) was proposed. It offloads the computation workload from passenger devices (PDs) to transportation infrastructures such as roadside units (RSUs) and base stations (BSs), called static fog nodes. It can also exploit the underutilized computation resources of nearby vehicles that can act as vehicular fog nodes (VFNs) and provide delay- and energy-aware computing services. However, the capacity planning and dimensioning of VFC, which come under a class of facility location problems (FLPs), is a challenging issue. The complexity arises from the spatio-temporal dynamics of vehicular traffic, varying resource demand from PD applications, and the mobility of VFNs. This paper proposes a multi-objective optimization model to investigate the facility location in VFC networks. The solutions to this model generate optimal VFC topologies pertaining to an optimized trade-off (Pareto front) between the service delay and energy consumption. Thus, to solve this model, we propose a hybrid Evolutionary Multi-Objective (EMO) algorithm called **S**warm **O**ptimized **N**on-dominated sorting **G**enetic algorithm (SONG). It combines the convergence and search efficiency of two popular EMO algorithms: the Non-dominated Sorting Genetic Algorithm (NSGA-II) and Speed-constrained Particle Swarm Optimization (SMPSO). First, we solve an example problem using the SONG algorithm to illustrate the delay–energy solution frontiers and plotted the corresponding layout topology. Subsequently, we evaluate the evolutionary performance of the SONG algorithm on real-world vehicular traces against three quality indicators: Hyper-Volume (HV), Inverted Generational Distance (IGD) and CPU delay gap. The empirical results show that SONG exhibits improved solution quality over the NSGA-II and SMPSO algorithms and hence can be utilized as a potential tool by the service providers for the planning and design of VFC networks.

## 1. Introduction

The integration of Internet of Things (IoT) systems with heterogeneous vehicular ad hoc networks (VANETs) has revitalized new capabilities in intelligent transportation systems (ITSs) [1]. Through machine-to-machine (M2M) communication protocols and standards, facilities such as RFID, WSN, GPS, etc., the IoT complements the evolution of ITS applications [2]. In fact, the future ITS is revolutionizing the transport system and autonomous mobility by the introduction of connected and autonomous vehicles (CAVs) with each vehicle having enormous capabilities in terms of storing, computing and networking (SCN). According to the worldwide CAV forecast from the International Data Corporation (IDC), the global market size of CAVs that are at upper levels of autonomy (L1≥) will rise from 31.4 million units during the period 2019–2020 to 54.2 million units during the period 2024–2025, reflecting a five-year Compound Annual Growth Rate (CAGR) of 11.5% https://www.idc.com/research/viewtoc.jsp?containerId=CA49221622, accessed on 20 October 2022). Feature by feature, the IoT adds novel capabilities for the smart, safe and secure operation of an incoming CAV fleet, a dramatic shift towards an increasingly autonomous future [3].

However, as a curse of volume, vast amounts of data are generated, prompting a delay- and energy-critical analysis and processing. Each CAV is to be equipped with at least 200 on-board sensor units (OBUs) for gathering the ambient information; hence, a galactic volume of sensed data is generated when it is running on the road [1]. According to preliminary estimates, a CAV generates approximately 30 terabytes of data every day. Furthermore, there will be a growing need for new services and frameworks that keep them connected while on the move for the rapid growth of connected mobility [4]. In such a scenario, efficiently processing the quality of service (QoS)-intensive CAV workload, extracting valuable information and making the correct decisions become critical issues.

Although cloud computing employs high-end servers called cloud data centers (CDCs) for processing vehicular data streams, they cause an intolerable delay and have a huge energy footprint [5]. Moreover, the presence of unstable connections in multi-user CAV environments degrades the overall quality of experience. For instance, many ITS applications need interactivity requiring a low response time and service delay for providing instant feedback [6]. In such scenarios, the control nodes must provide accurate signals for the steering system in milliseconds. In the case of a queuing delay or networking failure in cloud server, a vehicle may loose its intelligence, which may even lead to a crash. As a result, a fog computing (FC) model is proposed. Contrary to the cloud computing, fog deploys SCN capabilities into the intermediary in-network devices as well as in the CDCs, making a cloud-to-edge continuum. As a result, FC reduces the overall service delay for the VANET applications [7]. Moreover, since the data are processed and stored in the edge devices, the volume of data traffic transmitted through the network is also reduced [8].

**Figure 1:**
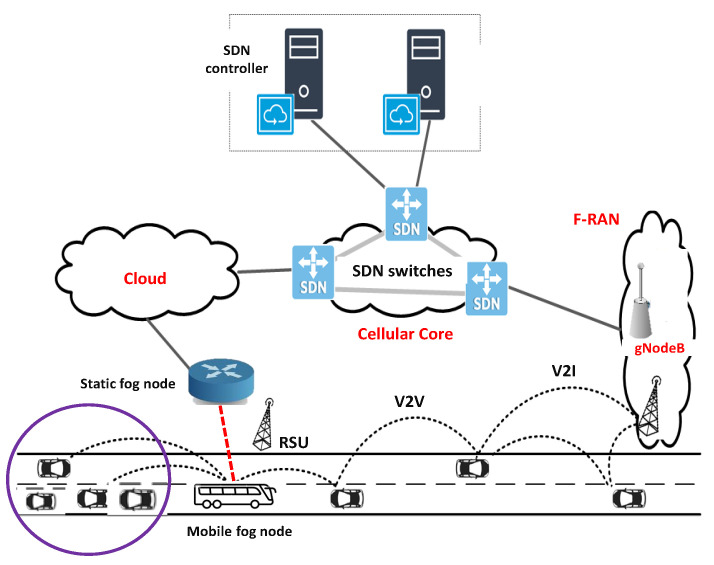
The hierarchical system architecture of VFC.

VFC is proposed as a special case of fog computing in VANETs [9]. It is an emerging paradigm that can potentially provide real-time and location-aware SCN services for a wide range of ITS applications [10]. The main idea of VFC is to employ the underutilized vehicular resources as active components of fog infrastructures and at the same time, enable the practical and effective use of conventional cloud resources [9,11]. VFC offers a context-aware, low latency, and energy-efficient SCN framework that leverages VANET users with a wide range of ITS applications [8]. It also enables on-road vehicles to make intelligent offloading decisions, instantly reacting and attuning to rapid changes in the ITS supply chain [12]. An overall architecture of VFC is given in Figure 1.

Because of its peculiar properties, the VFC has been attracting significant dedicated attention from the transportation industry and research communities in recent years [9]. The VFC infrastructure comprises fog nodes, both static (RSUs, BSs, etc.) and dynamic (heavy vehicles, taxis and buses) deployed at appropriate locations to provide computing services to the ITS users [12]. Although VFC architecture can provide ultra-low latency communications and a very low response time, deploying the architecture in the real world is a complex task [13]. The naive strategy to blindly place more and more resources is not practical because the corresponding cost will be very high. Thus, we need a mathematical framework to define the assignment of available resources across various architectural layers. In operation research, this is called an FLP. To be specific, a facility location model for VFC will deal with selecting optimal locations for installing a set of fog nodes, and the available channels for transferring vehicular data, by minimizing or maximizing some objectives. The solutions should satisfy the workload requirements with respect to a set of constraints defined over a given space [14]. The formal definition of a VFC FLP is given as [15]:

**Definition 1.**  *Facility Location Problem (FLP): Consider a bipartite graph with a bi-partition (F,V), where F and V represent the set of fog nodes and set of client vehicles, respectively. Suppose that the set $F=\{f_{1},f_{2}\ldots .\}$ denotes the potential facility locations, $c_{f}\geq 0$ denotes the deployment cost of a facility at location for f∈F with capacity $X_{f}^{max}\geq 0$, $c_{fl}\geq 0$ denotes the communication cost for each link between f∈F and v∈V, and $d_{v}\geq 0$ denotes a demand function for each client v∈V. Then, the goal of FLP is to find a subset $F_{1}\subseteq F$ and an assignment vector $\Phi :V\to F_{1}$ that maps requests from each client’s v∈V to appropriate facilities f∈F, such that the total communicating and processing cost is minimized. Note that this is the simplified definition sometimes known as simple FLP. However, we can generalize the depth of partition and make it a multi-level FLP [16].*

EMO algorithms are a class of algorithms inspired by a mechanism of natural evolution. They apply intelligent and approximate heuristics in order to search the optimal Pareto trade-off fronts. Compared to classical methods, preference-based algorithms include weighted sum methods [17], ϵ-constraint methods [18], and hierarchical methods [19], etc. The EMO algorithms offer a good balance between the Pareto optimality and search time efficiency. Hence, the EMO algorithms are becoming increasingly popular in the modeling and optimization of complex systems. A study reveals that a significant number of studies have used EMO algorithms to find the solution to resource management problems. These are commonly used to study the structural complexity of decentralized computing models such as cloud or fog computing [20], vehicular fog computing [12], etc. In fact, the survey performed by the authors of [21] found that, in approximately 18% of related studies, EMO algorithms were used to solve optimization problems such as VM allocation, VM migration, service placement, task offloading, and capacity planning, among others.

In this work, we present a multi-objective optimization model to formulate the FLP in VFC networks. The key objective of this model was to generate an optimal VFC topology that satisfies the service requirements of low-power and low-latency ITS applications. The objective function minimizes the average response delay and system energy consumption. The solutions to this model generate optimal decisions on where to install the fog nodes, how vehicle-generated requests are routed to these nodes, and the amount of storage–computation resources to be installed in each fog node. Essentially, we examine the suitability of different EMO algorithms to solve the FLP in VFC. The model is solved through one exact algorithm-weighted sum method in CPLEX solver, and two EMO algorithms; non-dominated sorting genetic algorithm-II (NSGA-II); and speed-constrained particle swarm optimization (SMPSO) algorithm. Motivated by the nature of solutions generated by these algorithms, we proposed a hybrid EMO algorithm called Swarm Optimized Non-dominated sorting Genetic Algorithm (SONG). Basically, SONG combines the convergence efficiency of NSGA-II [22] and search efficiency of SMPSO [23], and it gives better results in terms of solution quality. We measure the performance of the SONG algorithm by comparing with the baseline EMO algorithms against three baseline indicators, namely; hyper-volume (HV), inverted generational distance (IGD) and delay gap. The empirical results on real-world vehicular traces show that SONG exhibits better solution quality than the counterpart algorithms, and hence can be adapted as an efficient planning tool for planning and design of VFC networks. **The key highlights of this paper are given as follows:**

1. We formulate the FLP in VFC as a multi-objective optimization model combining two sub-problems: fog node placement (FNP) and fog node dimensioning (FND). The objective of this model is to minimize the average response delay and overall energy consumption, with an effort to attain Pareto-optimality.
2. We solve the model using an exact weighted-sum scalarization algorithm in the IBM ILOG CPLEX solver. The results obtained in this step are used as the baseline for EMO analysis. We also solved the model using two popular EMO algorithms: the NSGA-II and SMPSO algorithms. The nature of Pareto-fronts obtained in this step is the key driver for the development of a hybrid EMO algorithm.
3. We proposed a hybrid EMO algorithm called the Swarm Optimized Non-dominated Sorting Genetic algorithm (SONG) that combines the convergence efficiency of NSGA-II and the solution diversity of SMPSO. We compared and evaluated the efficiency of the SONG algorithm with that of weighted sum (CPLEX), NSGA-II, and SMPSO algorithms against three quality indicators; Hyper-Volume (HV), Inverted Generational Distance (IGD), and delay gap.
4. Through evaluation with real-world datasets, we showed that SONG exhibits a better solution quality than the three baseline algorithms, and can hence be utilized as an efficient planning tool for the planning and design of VFC networks.

The remainder of this paper is organized as follows. Section 2 gives a brief overview of the FLP and the motivation for conducting this research. Related works are given in Section 3. The system description and model formulations are provided in Section 4. Section 5 summarizes the exact and EMO algorithms for facility location in VFC. The proposed SONG algorithm is explained in Section 6. The evolutionary parameters of SONG algorithms are discussed in Section 7. Experimental and simulation setups are explained in Section 8. The results and discussions are given in Section 9. Finally, Section 10 concludes the paper.

## 2. Motivation

In distributed network environments, FLP refers to the identification of facilities for the deployment of storage and computing services [14]. The outputs of a facility location problem are: placement decisions regarding facilities that are placed at the potential locations; the links to be selected from an available set, and the amount of client workload that is to be transmitted across different architectural layers. It is a rational process that encompasses the topology design, network synthesis, and network realization [24]. The facility location model in VFC deals with selecting the optimal locations for installing a set of fog nodes, and the available channels for transferring vehicular data by minimizing or maximizing some objectives. The solutions should satisfy the workload requirements with respect to a set of constraints defined over a given space [25]. The constraints can be defined on available hardware, the fog node type and orientation, the channel bandwidth, etc. To be specific, it deals with the dimensioning and layout planning of VFC architectural components and generates a topology that optimizes parameters such as the response time, energy consumption, and cost of infrastructure, etc. [13]. Often, these parameters are conflicting, so the resulting topology must give an optimal balance (trade-off) between the network and equipment power consumption, interference and performance (e.g., in terms of throughput or latency).

However, due to the highly decentralized and mobile nature of fog nodes and context-aware computational demands of vehicular applications, the facility location in VFC is a complex task [6]. Here, the FLP can be partitioned into three sub-problems. The first sub-problem is an FNP that deals with selecting a subset of candidate nodes to act as fog nodes. FND is another sub-problem where the capacity levels of resources such as fog nodes, links and channels are estimated. The optimizing attributes for both these sub-problems are: the cost of the fog node, the weight (bandwidth) of communication links, the vehicular workload (# req/s) and other QoS parameters. The third sub-problem is computation offloading, i.e., the process of forwarding client requests to the appropriate nodes for execution. It is noteworthy that all these sub-problems are inter-dependent and are equally significant towards facility location in VFC networks. It has been theoretically proven that finding optimal solution for each of these individual sub-problems is NP-hard [26]. Obviously, a model that integrates all these sub-problems will also be NP-hard [16]. For an in-depth analysis of how facility location works in VFC, please refer to [25].

**Figure 2:**
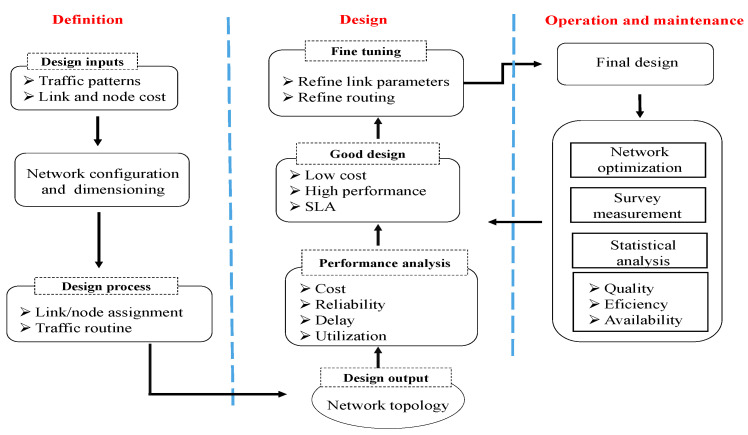
Steps involved in facility location in VFC.

As mentioned above, generating an optimal solution to facility location in VFC is a complex task due to many factors, e.g., the decentralized nature of VANETs, vehicle mobility, network size, the number and heterogeneous nature of the VFC hardware equipment(s), cloud–fog cooperation, etc. Therefore, it is critical for VFC network designers to adopt a structured methodology while formulating, designing, and planning VFC infrastructures [27]. Figure 2 shows a comprehensive framework for facility location in VFC networks. In the definition phase, the input data from the VANET environment are collected to conduct a requirement analysis. The information, for instance, the vehicular traffic patterns and traffic density, can be used to estimate the hardware type and cost, network capacity and QoS requirements. Obtaining real-world vehicular data is often complex and time-consuming. In that case, the inputs can be synthetically generated and then validated against a real-world ITS scenario [28].

In the network design phase, efficient dimensioning and layout planning algorithms are employed [29]. These involve activities such as the selection of various processing nodes, potential locations, the sizing and costing, etc. Depending on the design constraints, the output of this phase can be a routing topology guaranteeing optimal QoS requirements. Furthermore, these outputs are selectively fed as input to the optimization model. This generates optimal solutions that correspond to minimized service delay, network traffic, deployment cost and energy consumption. The final step is a performance evaluation that involves the sensitivity and statistical analysis of model outputs. From a designer’s perspective, the overall facility location framework must deliver the maximum network performance to ITS users, which can be targeted to meet the following key objectives:

- **Minimizing service delay:** Minimizing the service delay is persuaded to be the key performance objective in almost all networked architectures because a slight increase in the user-perceived delay might lead to a substantial revenue loss for the service providers. Since VFC services mainly target decentralized IoT devices and vehicles, an effective facility location framework should rigorously consider the latency requirements of ITS applications.
- **Minimizing energy consumption:** With a huge volume of service requests, the power consumption of powering up (and cooling down) CDC is soaring. It is thus necessary to study the energy management aspects of VFC design.
- **Minimizing capital expenditure (CAPEX):** Budgetary and monetary factors have always been a major concern for network designers. Therefore, it is crucial to minimize the total CAPEX involved in hardware purchasing, facility rental, link installation, etc.

It is observed that each of these objectives define sub-problems that are both inter-dependent as well as mutually conflicting [30]. For example, to minimize the service delay, a large number of fog nodes might be deployed, but this will increase the CAPEX. The energy consumption also gears-up proportionally. On the contrary, the CAPEX can be kept within budget by selecting fewer locations for fog placement. However, in that case, the client requests might not be served in the fog layer, but rather offloaded to distant cloud servers leading to a degradation in network performance and QoS. Considering all these factors, we formulate the FLP as a multi-objective optimization model where the objective function minimizes the average response time as well as system energy consumption. The optimal solutions called Pareto Fronts seek for the best compromise between the response time and energy consumption [31]. This leverages the VFC service providers as well as the network designers to assess the abilities of possible trade-off solutions, leading to intelligent network planning and design decisions.

## 3. Related Work

VFC extends fog computing to mobile VANETs, where the vehicles and RSU can both act as mobile fog nodes, thereby leveraging the full utilization of storage and computation resources. Considering the mobility of VANET infrastructures, the facility location and capacity planning in VFC recently received considerable attention. For example, in some papers, the VFC is studied from the viewpoint of delay optimization [9], energy optimization [32], utility maximization [33], cost minimization [6], etc.

The authors in [34] investigated the scenario wherein multiple vehicles formed a community and jointly shared computing resources to maximize their utility. The RSU acts as an orchestrator and takes charge of decision making for community formation and workload scheduling. According to [10], the RSU acts as both as a gateway to the remote CDC as well as a task scheduler.

Moreover, the use of EMO algorithms is becoming an emerging trend and has been widely adopted for resource management in decentralized architectures such as cloud, edge–fog computing, VFC, etc. EMO provides intelligent heuristics to solve a wide range of optimization problems such as VM allocation [20], data replica placement [35], federated clouds [36], among others. Studies that address the facility location in fog environments can be broadly categorized from numerous perspectives. We exhaustively reviewed the existing literature in fog server placement, identified the key highlights and presented the motivation for carrying out similar research, which is equally adaptive to the vehicular fog computing environments.

The first set of works minimizes network delays which are approximated through geospatial distant metrics between generation nodes and fog servers. For instance, the authors in [37], proposed an integer linear programming (ILP)-based service placement model that minimizes the hop-count between communicating nodes, thus reducing the communication latency and eventually the service latency. The bandwidth consumption between communicating services is implemented through a flow matrix. The goal of the architecture is to place the services in convenient for servers and allow these services to dynamically migrate according to the changing conditions of the network and status of the users. In [38], the authors proposed an semi-Markov decision process (SMDP)-based task offloading model for VFC considering the transmission delay, processing delay, available RSUs, and the variability feature of tasks and vehicle mobility into account. The model is solved using an iterative Bellman method where the objective is to maximize the long-term reward.

Souza et al. [39] proposed a QoS-aware service placement technique for cloud–fog scenarios in smart city environment. The model that minimizes the latency experienced by the services while guaranteeing the fulfillment of the capacity constraints. In [40], the authors employed priced timed Petri nets (PTPNs) models that allow the user to choose the available resources from a pre-allocated resource pool. The algorithm dynamically allocates the fog resources based on the predicted value of price-time weighted cost and credibility evaluation scheme. The authors in [8] have proposed a dynamic task allocation scheme that finds an optimal trade-off between service delay and quality loss constraints in VFC. The event-triggered task allocation problem is solved using a binary PSO algorithm and simulated in real-world vehicular mobility traces for video streaming and real-time object recognition tasks [41]. Considering the distributed capacity, the range and types of user applications and the mobility of IoT devices, Bittencourt et al. [42] analyzed the significance of concurrent, first-come first-served (FCFS), and delay-priority scheduling policies to improve the execution time in fog environments.

The authors in [43] employed modified NSGA-II to propose a multi-level resource scheduling model in the fog environment. To address the uneven distribution of the solution in the Pareto fronts in the standard NSGA, a modified crowding distance expression is defined that minimizes the service latency and improve the task execution stability. In [44], the authors employed the Tabu search for the joint optimization of data blocks placement and task scheduling in edge computing, with reduced computation delay and response time. Various parameters such as data block popularity, data storage capacity and replacement costs for data blocks, as well as replacement ratios of edge servers, are considered to calculate the value of each data block. Considering the containers as the lightweight resource unit for user services, an optimal placement scheme is presented that can avoid the repeated replacement of data blocks, thereby reducing the overall bandwidth overhead. Wang et al. [45] employed M/M/1 waiting queues to develop a latency-minimum offloading decision- and resource-allocation scheme for fog-enabled IoT networks. The optimization problem is solved using genetic simulated annealing-based algorithm to generate offloading decisions with improved convergence speed and quality.

The next thread of the literature focuses on minimizing the deployment costs of the servers while limiting maximal latency. For instance, the authors in [46] considered the client association, resource provisioning, task distribution, and VM placement as decision variables to develop a cost-effective optimization model for fog computing. The same set of variables were investigated in [47] to propose a cost optimal placement model fog-computing-supported medical cyber-physical system (MCPS). Urgaonkar et al. [48] modeled the dynamic service migration and workload scheduling in fog placement as a sequential Markov decision problem. The objective was to optimize operational costs while providing rigorous QoS guarantees. The same research group in [49] simulated the fog nodes as mobile micro-clouds and proposed a placement scheme based on an online approximation algorithm. The algorithm minimizes the average cost over time, and converges in polynomial time, leveraging the ability of predicting the future cost parameters with known accuracy. González et al. [50] employed the considered fog locations and their capacities, the user-to-fog association, and the number of deployed fog nodes as decision variables to model the fog placement as a mixed-integer linear programming (MILP) problem. The model is further solved using a hybrid simulated annealing algorithm, evaluated on a 5G desktop application leveraged with network function virtualization (NFV). A cost efficient data-driven VFC framework was proposed in a very recent work [6] where authors used both heuristic and integer linear programming (ILP) to solve the data-driven capacity planning problem. The objective is to minimize installation and operational expenses considering spatio-temporal variations in vehicular traffic and QoS constraints. A similar work was proposed in [31] that finds the optimal location of fog nodes in a green field scenario.

The third category of papers studies the impact of task and service placement strategies on the energy consumption of fog infrastructures. For example, the authors in [51] presented a placement scheme that minimizes the total energy consumption of the network, keeping the access delay within an acceptable threshold. The multi-objective optimization problem was solved using the particle swarm optimization (PSO) algorithm and the performance was evaluated on the real-world dataset of Shanghai Telecom’s base stations. In [52], the energy-sentient scheme for deploying flow-based IoT applications is proposed and formulated as a quadratic programming problem. The complexity of this model was handled by transforming it into to an MWIS problem via a co-location graph. Here, the mapping strategy seeks to minimize the total communication energy cost by merging the services and the co-locating neighboring services on the same node. In a recent work [53], the authors used reinforcement leaning to implement an energy-efficient vehicle scheduling scheme that ensures task offloading to stationary as well as mobile fog nodes.

Another perspective for fog placement is to optimize the trade-off between latency and energy consumption. Deng et al. [20] investigated the trade-off between power consumption and transmission delay in the fog–cloud computing system. A framework that obtains the optimal workload allocation is solved through sub-problem approximation approach. It demonstrates that, by sacrificing modest computation resources, fog computing can give a substantially improved performance over cloud computing. Mebrek et al. [54] studied the optimization problem of an energy-delay trade-off in fog-based IoT architectures using the Genetic Algorithm (GA) and Broadcast Incremental Power (BIP) algorithm. In [55], a computation offloading scheme was proposed that aimed to minimize the energy consumption and the task processing delay in the fog layer while increasing the network lifetime. The notion serves to distribute high computational tasks among several fog nodes and access points, such that the network lifetime increased, especially in mission-critical scenarios that consume much more network resources. Sarker et al. [56] employed multiple parallel deep neural networks to propose an delay-energy-optimized binary offloading scheme in fog computing. The decision outputs are then placed in a relay memory system to train and test all neural networks. A similar algorithm was proposed in [12], wherein task offloading in VFC was modeled as a Multi-Hop Computation Offloading (MHCO) framework. The model was solved using a modified differential evolution algorithm that optimizes both service delay and average energy consumption, and facilitates the allocation of tasks for both static (RSU) and vehicular fog nodes. Table 1 presents a summary of the literature in facility location or service placement in VFC and related architectures.

### Synthesis

From an extensive study of the recent literature, we concluded that the algorithms to solve FLPs can be categorized as: exact methods and approximate or EMO methods. Most exact algorithms combine multiple objectives into one single objective. A common advantage of such methods is that, for every weight assigned to the objectives, the translated problem is as difficult as a single-objective optimization problem. This makes the exact methods simple to implement. However, due to the presence of discrete structures in the instances of FLP, it is not sufficient to aggregate multiple objectives using a weight vector. Because of NP-hardness, there will be solution called non-supported efficient (NE) solutions which are not optimal for any weighted sum of objectives.

EMO algorithms come under approximate resolution methods, commonly termed meta-heuristic algorithms. These algorithms yield a good trade-off between the solution set quality, CPU time, and memory [22]. They can be classified based on the principle of search directions and principle of dominance, where they take advantage of the information carried by a population of solutions using the notion of dominance. On the contrary to exact algorithms where only one individual is attracted to the non-dominated frontier, in EMO, all the population contributes to the evolution process. Thus, by maintaining a solution pool, then these methods can search for multiple efficient solution points in parallel via self-adaptation and cooperation. Such characteristics make EMO algorithms suitable for solving FLP in VFC [26]. Driven by this motivation, in what follows, we discuss the popular EMO algorithms that solve the multi-objective versions of an FLP. We also propose a new EMO algorithm named SONG, which is characteristically a hybrid version of NSGA-II and SMPSO. We performed extensive experimentation on a wide set of inputs to demonstrate the efficiency of SONG. In particular, we compared the performance of SONG with that of NSGA-II and SMPSO, and showed that SONG offers a better balance between the Pareto optimality and computation time efficiency.

## 4. System Description and Problem Formulation

The system model is explained in detail in this section. We first introduced the scenario and then described the procedure of offloading a task in VFC according to the 802.11p standard [38]. The VANET scenario considered in this paper is modeled as a connected undirected graph G{V(.),E(w)}. The vertices in set V(.) include a set of vehicles $\left\{v_{1},v_{2},\ldots v_{V}\right\}$, a set of fog nodes $F=\{f_{1},f_{2},\ldots f_{F}\}$ and a cloud server (C). The vehicles moving on highway lanes form a VFC system equipped with seamless connectivity to an upper fog–cloud layer via BS or RSU. The function E(w) denotes the set of edges having weights $w=\{w_{1},w_{2}\ldots \}$. Here, each weight $w_{i}$ represents the network usage descriptor (NUD) between each pair of vertices in *V*(.). In our analysis, we model $w_{i}$ by a triple $(r_{n2n},\omega_{n2n},\tau_{n2n}),$ where $r_{n2n}$ denotes the transmission rate between two nodes, $\omega_{n2n}$ denotes the link bandwidth between two nodes and $\tau_{n2n}$ denotes the end-to-end delay. The number of passengers residing inside each vehicle *v* varies randomly between 0 and $P_{v}$, with one PD per person. Hence, the total number of PDs in a VFC system (cluster) can be given as $N=\sum_{v=1}^{V}P_{v}$.

A vehicle joining or leaving the VFC system according to a Poisson process with an arrival and departure rate $\lambda_{v}$ and $\mu_{v}$, respectively. The vehicles adopted the 802.11p communication standard to transfer data/request through one-hop communication [8]. Each vehicle knows the availability fog nodes by communicating with each other through handshaking [12]. When the PD has a task to offload, the task offloading module makes a decision to offload it onto appropriate fog nodes. On the basis of the estimation of available resources, e.g., memory, vCPU, VM, bandwidth, etc., the host vehicle will either accept the task with a probability $\Pi_{v}$ or offload to a nearby fog node or to remote CDC. For the sake of simplicity, it is assumed that fog nodes, i.e., taxis as well as RSU are connected through a strong backhaul network [27].

**Figure 3:**
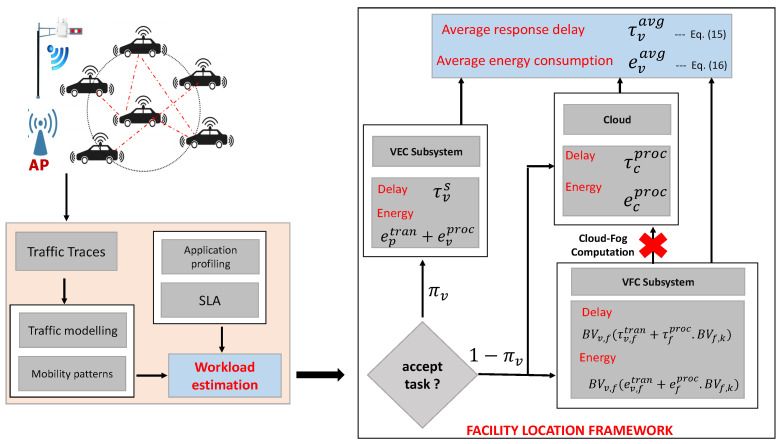
An overall framework of power consumption-delay trade-off in a VFC system.

On the basis of the assumptions discussed above, we formulated the model for average response delay $\tau_{sys}^{avg}$ and average energy consumption $e_{sys}^{avg}$ per vehicular application, for the different scenarios of facility location in VFC. The overall framework is given in Figure 3. The key symbols and notations used for the model are summarized in Table 2.

**Decision Variables:**

- A probabilistic variable $\Pi_{v}$ representing whether the host vehicle *v* accepts the PD request.
- Binary variable $BV_{v,f}$ representing cloud–fog offloading decision (where to offload), i.e.,
  (1) $$BV_{v,f}=\left\{\begin{array}{c} 1\;task\;from\;vehicle\;v\;is\;offloaded\;to\;fog\;node\;f\\ 0\;to\;cloud \end{array}\right.$$
- The RSUs or taxis/buses are both potential candidates to act as a fog node. For a time interval, a subset of these nodes will be selected for processing the PD workloads. The binary variable $BV_{f,k}$ denotes whether an RSU or a taxi/bus *k* is selected as a fog node *f*.
  (2) $$BV_{f,k}=\left\{\begin{array}{c} 1\;if\;RSU\;or\;taxi/bus\;k\;is\;selected\;as\;fog\;node\;f\\ 0\;not\;selected \end{array}\right.$$

**Definition 2.**  *Task: A task $T_{v}$ generated from a vehicle v can be defined by a triplet $\langle \sigma_{v},F_{v},\tau_{v}^{max}\rangle$, where $\sigma_{v}$ is the size of task in bytes, $F_{v}$ denotes the number of CPU cycles needed to process the task $T_{v}$[27] and $\tau_{v}^{max}$ denotes the delay constraint for the vehicular application that generates the task $T_{v}$.*

**Definition 3.**  *Response delay: The response delay $\tau_{v}^{avg}$ for a task $T_{v}$ is the time required to serve it, i.e., the time interval between the moment when a client vehicle makes a service request and when it receives the response (result) for that request.*

### 4.1. Delay and Energy Formulation

- **Case I: When vehicle *v* accepts the PD request:** Generally, a vehicle pools requests from its resident PDs, and each task is assumed to be computationally intensive and mutually independent. In case a vehicle *v* accepts the request coming from PD p∈P with an arrival rate $r_{p}^{a}$, the overall workload $r_{v}^{a}$ for vehicle *v* can be given as:
  (3) $$r_{v}^{a}=\sum_{p=1}^{P}r_{p}^{a}\;\forall p\in P\;\forall vs.\in V$$
  Modeling the vehicular edge computation as an M/M/1 queue, the service time when *v* processes a task from a PD *p* can be expressed as:
  (4) $$\tau_{v}^{s}=\frac{\sum_{p\in P}\tau_{p}^{s}}{\left|V\right|}=\frac{1}{\left|V\right|}\sum_{p=1}^{N}\frac{1}{r_{v}^{cap}\left(1-{POF}_{v}\right)-\Pi_{v}\cdot r_{v}^{a}}$$
  where $r_{v}^{cap}$ denotes the processing capability of the vehicle *v*, and $POF_{v}$ denotes the normalized workload on vehicle *v*, i.e., the fraction of processing a resource that is currently involved in serving other tasks. If $POF_{v}=1$, then the CPU is fully occupied and hence the requests are offloaded either onto fog nodes or the cloud. Every time a vehicle is on the highway, it is processing some kernel tasks, therefore,
  (5) $$0\leq POF_{v}^{V}<1$$
  In our VFC framework, the client requests are forwarded via dedicated short-range communication (DSRC) or cellular V2X (C-V2X [8], hence the energy required by PD *p* to transmit a task of size $\sigma_{v}$ over a channel bandwidth $\omega_{p,v}$ is given by [57]:
  (6) $$e_{p}^{tran}=\frac{P_{p}^{tran}\cdot \sigma_{v}}{\omega_{p,v}}$$
  where $P_{p}^{tran}$ is the transmission power of PD *p*. The corresponding energy required by vehicle *v* to execute this task is given by [27]:
  (7) $$e_{v}^{proc}=F_{v}\cdot \beta_{v}$$
  where $\beta_{v}$ denotes the energy consumption (in energy units) per unit CPU cycle of task $T_{v}$.
- **Case II: When the vehicle *v* offloads the PD request to the fog node:** Because of a limited processing capability, the host vehicle frequently fails to execute concurrent PD requests; hence, it is forwarded to a proximate fog node. Considering a reliable communication link of bandwidth $\omega_{v,f}$, the time required to offload the input request of size $\sigma_{v}$ into fog node *f* is given by:
  (8) $$\tau_{v}^{tran}=\frac{r_{f}^{a}\cdot \sigma_{v}}{\omega_{v,f}}$$
  Similarly, the processing delay due to processing at fog node *f* with computing capacity $C_{f}$ is given by:
  (9) $$\tau_{f}^{proc}=\left\lceil \frac{F_{v}}{C_{f}}\right\rceil$$
  The energy consumed while forwarding a task of size $\sigma_{v}$ is given by:
  (10) $$e_{v,f}^{tran}=\frac{\alpha_{v}\cdot \sigma_{v}}{\omega_{v,f}}$$
  where $\alpha_{v}$ depends on the communication channel over which the task is offloaded. For 5G-enabled V2X communication, we consider $\alpha_{v}=2605$ mJ/s [58]. The energy consumed by fog node *f* to process task $\sigma_{v}$ can be expressed as:
  (11) $$e_{f}^{proc}=E_{f}\cdot r_{f}^{a}\cdot \sigma_{v}$$
  where $E_{f}$ and $r_{f}^{a}$ represents the energy consumption per CPU cycle of fog node *f*, and the number of CPU cycles required to process one bit of task $T_{f}$, respectively.
- **Case III: When vehicle *v* offloads the PD request to CDC:** The time for the cloud layer consists of two parts. First, the transmission time for dispatching and processing time at the cloud server. Assuming the vehicle-cloud channel has infinite bandwidth, the transmission time can be considered constant [30]. Realizing the cloud servers as an M/M/∞ queue with a service rate $r_{c}^{s}$, the processing time is given by:
  (12) $$\tau_{c}^{proc}=\frac{1}{r_{c}^{s}}$$
  Assuming that each server *c* hosts a homogeneous VM with an identical CPU frequency, then the energy consumption can be approximated by a nonlinear function of CPU frequency [20], i.e.,
  (13) $$\eta_{c}=A_{c}\cdot \eta_{c}^{u}+B_{c}$$
  where *u* lies between 2.5 and 3 [20]. In order to cope with the workload surge, the CDCs are enabled with power-saving modes wherein more servers are either powered ON or switched OFF dynamically. In that case, the energy consumption is given as:
  (14) $$e_{c}^{proc}=BV_{c}\cdot n_{c}\cdot \eta_{c}$$
  where the binary decision variable $BV_{c}$ reflects the ON/OFF state of the server *c* (0-OFF, 1-ON), and $n_{c}$ denotes the number of machines that are currently turned ON. For the sake of keeping the model simple, we assume that the cloud servers are always ON (i.e., $BV_{c}=1$).

Integrating Equations (4), (8), (9) and (12), we obtain the average response delay $\tau_{v}^{avg}$ as:

(15) $$\begin{array}{c} \tau_{v}^{avg}=\frac{\sum_{v\in V}\sum_{p\in P}\tau_{v}}{\left|V\right|}=\\ \frac{1}{\left|V\right|}\cdot \{\overset{\mathrm{PD}\;\mathrm{to}\;\mathrm{vehicle}}{\overset{︷}{\Pi_{v}\cdot \tau_{v}^{s}}}+\overset{\mathrm{vehicle}\;\mathrm{to}\;\mathrm{fog}\;}{\overset{︷}{\left(1-\Pi_{v}\right)\cdot [BV_{v,f}\left(\tau_{v,f}^{tran}+\tau_{f}^{proc}\cdot BV_{f,k}\right)}}\\ +\underset{\mathrm{vehicle}\;\mathrm{to}\;\mathrm{cloud}}{\underset{︸}{\left(1-BV_{v,f}\right)\cdot \tau_{c}^{proc}\}]}}\}\;\forall p\in P \end{array}$$

Similarly, from Equations (6), (7), (10), (11) and (14),

(16) $$\begin{array}{c} e_{v}^{avg}=\frac{\sum_{v\in V}\sum_{p\in P}e_{sys}}{\left|V\right|}=\\ \;\frac{1}{\left|V\right|}\cdot \{\overset{\mathrm{PD}\;\mathrm{to}\;\mathrm{vehicle}}{\overset{︷}{\Pi_{v}\cdot \left(e_{p}^{tran}+e_{p}^{proc}\right)}}+\overset{\mathrm{vehicle}\;\mathrm{to}\;\mathrm{fog}\;}{\overset{︷}{\left(1-\Pi_{v}\right)\cdot [BV_{v,f}\cdot \left(e_{v,f}^{tran}+e_{f}^{proc}\cdot BV_{f,k}\right)}}\\ +\underset{\mathrm{vehicle}\;\mathrm{to}\;\mathrm{cloud}}{\underset{︸}{\left(1-BV_{v,f}\right)\cdot e_{c}^{proc}]}}\}\;\forall p\in P \end{array}$$

### 4.2. Optimization Model

The overall goal of the proposed facility location model is to minimize the average response delay $\tau_{v}^{avg}$ and energy consumption $e_{v}^{avg}$ of the VFC components. Hence, the optimization problem can be formulated as:

(17) $$\begin{array}{ccccc} \; & P1\; & \underset{\forall vs.,\forall f,\forall l}{\mathrm{minimize}}\; & \; & F(\tau_{v}^{avg},e_{v}^{avg})\\ \; & \mathrm{Subject}\mathrm{to}\mathrm{constraints}: \end{array}$$

(18) $$r_{v}^{cap}\left(1-{POF}_{v}\right)>\Pi_{v}\cdot r_{v}^{a}$$

(19) $$r_{c}^{s}>r_{f}^{s}$$

(20) $$\tau_{avg}<\tau_{v}^{max}$$

(21) $$\sum_{f\in F}BV_{v,f}=1\;\forall v\in V$$

(22) $$\sum_{v\in V}BV_{v,f}=BV_{f,k}\;\forall f\in F$$

(23) $$\sum_{f\in F}BV_{v,f}\cdot r_{f}^{a}<C_{f}\;\forall v\in V$$

(24) $$\sum_{f\in F}BV_{f,k}\leq 1\;\forall k\in K$$

(25) $$BV_{v,f}-\sum_{f\in F}BV_{f,k}\leq 0\;\forall k\in K$$

Constraint (18) says that the workload at vehicle *v* must not exceed its maximum processing capacity. Constraint (19) is an integrity constraint that keeps the service rate of CDCs much higher than that of fog nodes. Constraint (20) is a QoS constraint which the response delay for any task must not exceed the delay threshold specified for that vehicular application. Equation (21) is a vehicular assignment constraint that assumes the workload generated from each vehicle v∈V to be atomic. At every time-slot, a task is either offloaded for fog level processing or to the cloud, but not to both. Likewise, constraint (22) says that the vehicle *v* should send its workload to one and only fog node. Constraint (23) ensures that the total workload of a fog node should not exceed its processing capacity. For simplicity, we use the constraint (24) as a node uniqueness constraint that limits each location k∈K to accommodate at most one fog node f∈F. This is realistic because an RSU can act as a single fog node only. However, it is also possible to install additional servers at the RSU site, when it will be hosting multiple fog nodes. Moreover, it also means that a VFN will not be selected in the range exclusive for RSU. Finally, constraint (25) guarantees that each vehicle *v* can only offload to a location *k* iff a fog node *f* is installed there.

**Remark 1.**  *As mentioned in the system description, we abstract the weight vector E(w) through an NUD. It represents the resource usage between each facility in the VFC facility location. Since NUD is transparent to the mathematical solver and algorithms, it can be modified beforehand to meet specific design and dimensioning requirements. For example, if a specific passenger does not want to upload their data to a specific node due to security concerns or policy issues, then we may label the corresponding PD-fog node link as infinite. In that case, their data will never be forwarded to this adverse fog node and hence, a security requirement will be safeguarded.*

### 4.3. Complexity Analysis

It can be observed that the multi-optimization model P1 has two conflicting objectives. A trivial strategy is to handle each objective sequentially by translating them into independent single objective sub-problems. However, this approach does not explore the interconnections and overlaps between sub-problems, and hence the solutions may suffer from a local optimum. Thus, it is preferable to employ a global approach, i.e., solve all sub-problems simultaneously.

For complexity analysis, let us relax and make three versions of P1: SP1, SP2 and SP3, from three perspectives. SP1 aims to minimize the response time and SP2 aims to minimize the energy consumption, while SP3 is a weighted combination of both SP1 and SP2, i.e.,

(26) $$\begin{array}{ccccc} \; & \mathrm{SP}1\; & \underset{\forall v,\forall f,\forall l}{\mathrm{minimize}}\; & \; & F_{1}\left(\tau_{avg}\right)\\ \; & \mathrm{Subject}\mathrm{to}\mathrm{constraints}:\;e_{sys}\leq e_{min} \end{array}$$

(27) $$\begin{array}{ccccc} \; & \mathrm{SP}2\; & \underset{\forall v,\forall f,\forall l}{\mathrm{minimize}}\; & \; & F_{2}\left(e_{sys}\right)\\ \; & \mathrm{Subject}\mathrm{to}\mathrm{constraints}:\;\tau_{sys}\leq \tau_{min} \end{array}$$

(28) $$\begin{array}{cc} \; & \mathrm{SP}3\\ \min_{\forall v,\forall f,\forall l}\; & F\left(\tau_{avg},e_{sys}\right)=C_{1}\cdot \sum_{f\in F}e_{sys}+C_{2}\cdot \sum_{v\in V}\tau_{avg}\\ \; & \mathrm{Subject}\mathrm{to}\mathrm{constraints}:\;18–25 \end{array}$$

**Definition 4.**  *k-median clustering problem: Consider a bipartite graph with a bi-partition (F,V) where F represents a set of fog nodes and V is a set of clients. Let k be a positive integer that specifies the number of nodes that are allowed to be deployed (e.g., may be limited by budgetary constraints). Suppose that the set $w_{vf}$ denotes the cost (weight) of connection(s) between vehicle v∈V to fog node f∈F. Then, the goal of the k-median clustering problem is to select k representatives of F to minimize the total connecting cost. Mathematically,*

*k*-MEDIAN CLUSTERING

*Input:* Finite set *F* and integer *k*

*Output:*$F^{*}\subset F$ with $|F^{*}|=k$

*Objective:* minimum cost$\left(F^{*}\right)=\sum_{x\in F}\rho \left(x,F^{*}\right)$

**Theorem 1.**  *SP1 is NP-hard.*

**Proof.**  To approximate the hardness of SP1, we relax certain constraints. For instance, assume that we have prior information about the number of fog nodes *f* that can be deployed and are available. We also relax the capacity constraint for nodes at each location l∈L. In that case, the decision problem SP1 of finding an assignment vector from vehicle v∈V to fog node f∈F that minimizes the total response delay can be reduced to a *k*-median clustering problem. It is well known that the k-median clustering problem is NP-complete [16]. Since an instance of SP1 can be reduced to a k-median clustering problem (by definition 5.1), it is therefore also NP-hard.    □

**Theorem 2.**  *SP2 is NP-hard.*

**Proof.**  Upon adopting a relaxation strategy similar to that for SP1, SP2 becomes an instance of the traveling salesman problem (TSP) with pre-determined initial and final locations. Since, TSP is known to be NP-hard, SP2 is also NP-hard.    □

**Theorem 3.**  *SP3 is NP-hard.*

**Proof.**  If we carefully observe the SP3 and the definition of FLP in Section I, we will find that FLP is an instance of SP3 (provided with some relaxation). Since finding an optimal solution to FLP on general graphs is NP-hard, then SP3 is also NP-hard.    □

## 5. Algorithms for Facility Location in VFC

### 5.1. Exact Algorithms

It can be observed from Equations (15) and (16) that the optimization model P1 is a mixed-integer nonlinear programming (MINLP) problem with two conflicting objectives, which is NP-hard [59]. Different categories of algorithms have been proposed in the literature to solve such problems, e.g., exact methods and approximate or heuristics, nature-inspired EMO, etc. The exact methods can be further categorized into several classes, viz. labeling algorithms, greedy algorithm, scalarization methods, two phase methods, and branch and bound algorithms. Most of the algorithms in this class combine multiple objectives into one single objective. A common advantage of such methods is that for every weight combinations of $\alpha_{1}$ and $\alpha_{2}$, defined for the objectives, the translated problem is as difficult as a single-objective FLP.

The weighted sum is a popular scalarization method that has been widely adopted for facility location problems. In the weighted sum approach, multiple objective functions are combined into a single objective function, where individual objectives are assigned a certain weight between 0 and 1. In other words, multiple objectives $F_{1}\left(x\right)$, $F_{2}\left(x\right)$, $F_{3}\left(x\right)$,……$F_{n}\left(x\right)$ in a multi-objective decision problem are translated into a weighted single objective function given as [60]:

(29) $$\begin{array}{cc} \; & \mathrm{minimize}\{\alpha_{1}F_{1}\left(x\right)++\alpha_{2}F_{2}\left(x\right)+\ldots \ldots +\alpha_{n}F_{n}\left(x\right)\}\\ \; & \mathrm{Such}\mathrm{that}: \end{array}$$

$$0\leq \alpha_{i}\leq 1$$

$$\sum_{i=1}^{n}\alpha_{i}=1$$

where $\alpha_{i}$ is the weight factor assigned to each objective to capturing the relative importance of a given objective $F_{i}\left(x\right)$. Assigning a higher (lower) weight places more or (less) emphasis on a specific objective. Formula (29) is used to minimize or maximize the parametric sum of all objective functions. By varying the weight vector, all supported efficient (SE) solutions can be found.

However, the success of exact algorithms to solve FLPs entirely depends on the availability of good lower and upper bounds *ℓ* and *u*, respectively, i.e., $\ell \leq X^{*}\leq u$. Here, $X^{*}$ is the optimal value (solution) of the optimization problem. Often, it is not sufficient to aggregate multiple objectives through weighted sum methods due to the presence of discrete and conflicting structures in the FLPs. Furthermore, due to their NP-hard nature, there will be solutions called Non-supported efficient (NE) solutions which are not optimal for any combination of weight factors. For a non-convex function, it is difficult to obtain the weight vectors $\alpha_{i}$ that will generate Pareto-fronts lying in a desired region of objective space. Additionally, it is unrealistic to extend the exact methods to a multi-objective scenario or have more than a few hundred variables.

### 5.2. Evolutionary Multi-Objective (EMO) Algorithms

Evolutionary or meta-heuristic algorithms are a class of algorithms inspired by natural evolution mechanisms. Compared to exact and simple heuristics, they provide a more efficient search for larger and complicated problems, and yield a good trade-off between solution set quality, time and memory [22]. In general, evolutionary algorithms are broadly categorized based on the principle of search directions and principle of dominance, i.e., information is carried forward by a population (swarm) of solutions [61]. In contrast to exact methods where only a single individual is attracted to the non-dominated frontier, all the population in the solution pool contributes to the evolution process. Essentially, by maintaining a solution pool, the meta-heuristics methods can search for multiple efficient solution points in parallel via self-adaptation (independent evolution and assortment) and cooperation (the exchange of information between individuals) to improve the solution quality [26]. Such characteristics make population-based approximation algorithms suitable for multi-objective FLPs in VFC [26,59].

#### 5.2.1. Genetic Algorithm

   Genetic Algorithm (GA) [62] is the first class of population-based optimization algorithms proposed to solve FLPs. GA iteratively evolves a set of encoded solutions using three key operations, viz. *reproduction, crossover* and *mutation* in order to converge to an optimal solution [22]. At each iteration, a fitness function is defined to quantify the optimality of solutions where the parent/child selection is based on individual fitness. The *fitness function* ensures that better solutions persist in future generations and weaker solutions are eliminated. In other words, the higher the fitness level (value), the greater the chance is that the solution contributes to creating solutions in the subsequent generation. This process halts when the predefined conditions (e.g., maximum number of iterations) are met.

Normally, a single-objective variant of GA [62] combines the multiple objective functions into a single-composite function. It does so by weighting the objectives with a weight vector, as in the case of an exact algorithm discussed in Section 5.1. An alternate strategy is to keep only one among them as the main objective in the optimization model and keeping rest as varying constraints. To obtain Pareto-optimal solutions, both of these techniques require several runs. However, because of its ability to concurrently search for the different regions of a solution space, a single-objective GA can be transformed into a multi-objective GA (MOGA), which finds a set of Pareto optimal solutions in a single run. Since most multi-objective GA algorithms do not require the user to prioritize, scale, or weight any of the objective(s), they are among the most frequently used meta-heuristics scheme for solving FLPs, even with conflicting objectives [26].

#### 5.2.2. Non-Dominated Sorting Genetic Algorithm (NSGA-II)

The key objectives in the design of MOGA algorithms are:

- Guiding the search process towards the Pareto set through fitness assignment and offspring selection.
- Preserving the diversity of Pareto fronts.
- Retaining the best chromosomes for subsequent generations (elitism).
- Decreasing the computational time (e.g., CPU time).

The NSGA algorithm was among the first algorithms of the class of multi-objective GA [22]. The earliest version of this algorithm received numerous criticisms because it has high computational complexity $\left[O\left(AB^{3}\right)\right]$ for a non-dominated sorting procedure (where *A* denotes the number of objectives and *B* represents the size of the population). Another prominent issue with NSGA is the lack of elitism which leads to the loss of quality solutions. Additionally, NSGA requires specifically sharing the parameter explicitly to ensure diversity preservation. In order to address all such shortcomings, Deb et al. [22] proposed an improvement over NSGA which is faster than NSGA, which is termed NSGA-II. NSGA-II has the computational complexity of $\left[O\left(AB^{2}\right)\right]$. NSGA-II uses the identical method as the generic GA with the distinction that the latter makes use of an elitist ranking procedure (i.e., the non-dominated sorting procedure). The NSGA-II preserves the best population inside the latest solution pool utilizing the fast ranking procedure. In Algorithm 1, we explain the steps for the locate the facility using the NSGA-II algorithm.

**Figure 4:**
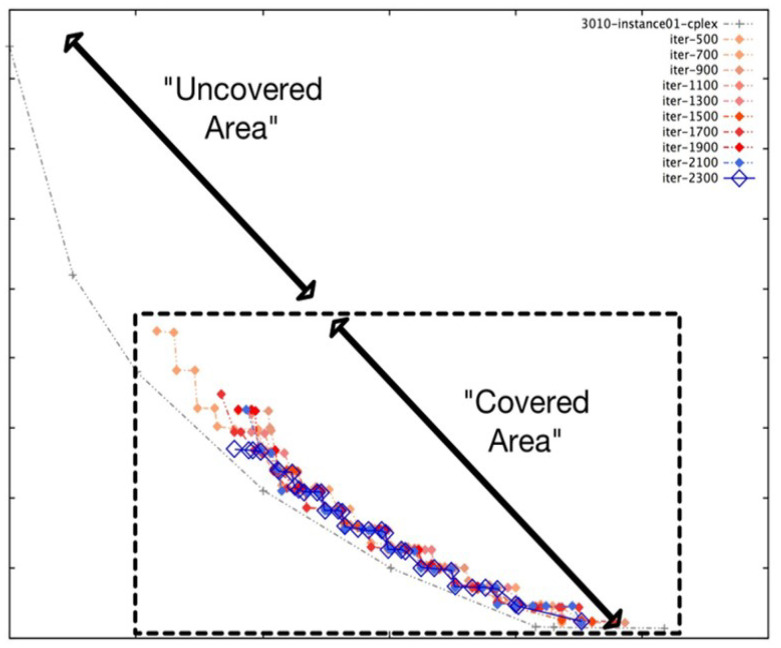
NSGA-II algorithm struggles to cover the whole Pareto front.

Although NSGA-II efficiently handles continuous multi-objective problems, it does not appear to be ideal for service placement problems due to its characteristic discrete nature [25]. For instance, in some specific FLPs, as shown in Figure 4, we found that although NSGA-II converges quickly towards the Pareto fronts, the search space is irregularly distributed. Nonetheless, in many iterations, the NSGA-II struggles to cover the whole Pareto front. Actually, this shortcoming stems from the ranking process (between line 5 and 7) employed in NSGA-II, where the concentrated effect of a non-dominated sorting procedure harms the diversity in NSGA-II’s solution fronts. Even if the mutation index or crossover index are varied, no meaningful improvement is achieved. Additionally, the non-dominated sorting procedure also affects the diversity of solutions in NSGA-II. Although such shortcomings can be overcome by an elitism strategy, the temporal complexity of evaluating the elitist sets is extremely high, which might be a major issue for large-scale FLPs.
**Algorithm 1** NSGA-II Algorithm for facility location in VFC
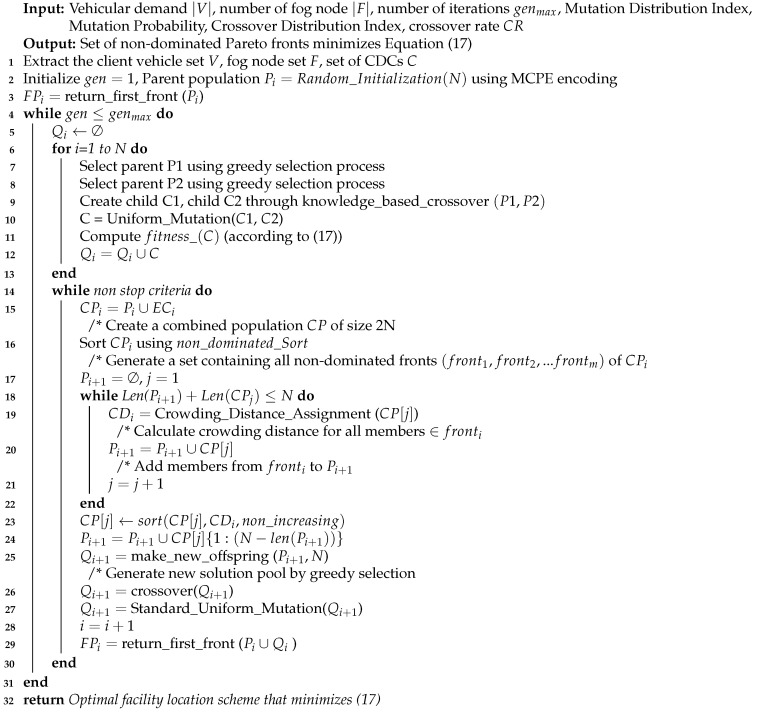


#### 5.2.3. Particle Swarm Optimization (PSO)

Another class of evolutionary algorithms given by Kennedy et al. called particle swarm optimization (PSO) imitates the social behavior of bird flocking or fish schooling [61]. Basically, the PSO imitates a swarm of *S* potential solutions called particles which move through a *D*-dimensional solution space, as they hunt for a global optimum.

PSO is a stochastic population-based algorithm that is easier to implement than GA, as this latter requires excessive effort to tune a large number of parameters. Additionally, PSO exploits a larger search space than GA [63]. Moreover, the particles in PSO have a memory term which is important for the algorithm. In PSO, each particle *p* is allocated a random position $x_{p}$ and a velocity $v_{p}\left(p=1,2\ldots |S|\right)$. In every iteration, the particle adjusts its velocity according to the previous best ($x_{pp}$) and global best solution (leader, $x_{gp}$). The position vector of the particle *p* is updated at time-step *t* according to the equation:

(30) $$x_{p}^{t}=x_{p}^{t-1}+v_{p}^{t}$$

where the velocity function $v_{p}^{t}$ is given by Equation (28), where C1 and C2 are two positive learning factors (also referred to as trust parameters), $r_{1}$, $r_{2}$ are random vectors.

(31) $$v_{p}^{t}=\overset{\mathrm{Inertia}}{\overset{︷}{w\cdot v_{p}^{t-1}}}+\underset{\mathrm{Personal}\;\mathrm{influence}}{\underset{︸}{C_{1}\cdot r_{1}\cdot \left(x_{pp}-x_{p}\right)}}+\underset{\mathrm{Social}\;\mathrm{influence}}{\underset{︸}{C_{2}\cdot r_{2}\cdot \left(x_{gp}-x_{p}\right)}}$$

However, for MOCO problems, the constrained-based fitness function for two solutions $Y_{i}$ and $Y_{j}$ need to be redefined, so that it fits in the multi-objective context. Here, a solution $Y_{i}$ is said to “*constrain-dominate*” over a solution (particle) $Y_{j}$ (i.e., $Y_{i}≺Y_{j})$, if any of the following conditions are true:

- Solution $Y_{i}$ is feasible and solution $Y_{j}$ is not.
- Solution $Y_{i}$ and $Y_{j}$ are both infeasible, but solution $Y_{i}$ might have a smaller constraint violation (which can be computed by adding the normalized violation of all constraints).
- Both solutions $Y_{i}$ and $Y_{j}$ are feasible and solution $Y_{i}$ dominates solution $Y_{j}$ in the sense of Pareto dominance.

#### 5.2.4. Speed-Constrained Multi-Objective PSO (SMPSO)

     As the name suggests, SMPSO is an improved version of the PSO algorithm [23], which is distinguished by its use of a velocity-limiting procedure, for generating new effective particle positions in cases where the velocity exceeds a limit. Additionally, SMPSO employs a turbulence factor over γ percent of the particles (in place of polynomial mutation) and an external ranked list to store the non-dominated Pareto fronts obtained during the search process. This makes SMPSO fast and more accurate than generic PSO. The pseudo code of SMPSO-based facility location is given in Algorithm 2. The key difference between the SMPSO and generic PSO is that, instead of controlling the velocity step size with upper- and lower-valued parameters, SMPSO adopts a constriction coefficient *ℵ* on the resulting velocity given as:

(32) $$ℵ=\frac{2}{2-\varphi -\sqrt{\varphi^{2}-4\varphi }}$$

where

$$\partial =\left\{\begin{array}{c} C_{1}+C_{2}\;if\;C_{1}+C_{2}>4\\ 0\;if\;C_{1}+C_{2}\leq 4 \end{array}\right.$$

**Algorithm 2** SMPSO Algorithm for facility location in VFC

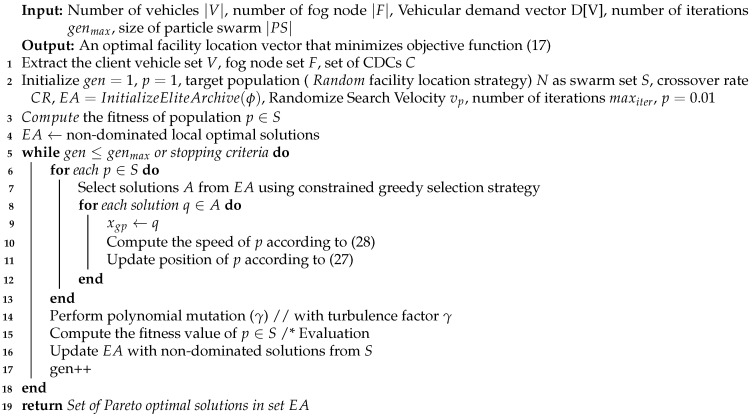



Furthermore, the accumulated particle velocity (resulting value) of each particle *p* is further bounded by constriction equation.

$$v_{p}^{t}=\left\{\begin{array}{c} \partial \;v_{p}^{t}>\partial \\ -\partial \;if\;v_{p}^{t}\leq \partial \\ v_{p}^{t}\;otherwise \end{array}\right.$$

where

$$\partial =\frac{upper\text{\_}lim_{p}-lower\text{\_}lim_{p}}{2}$$

**Figure 5:**
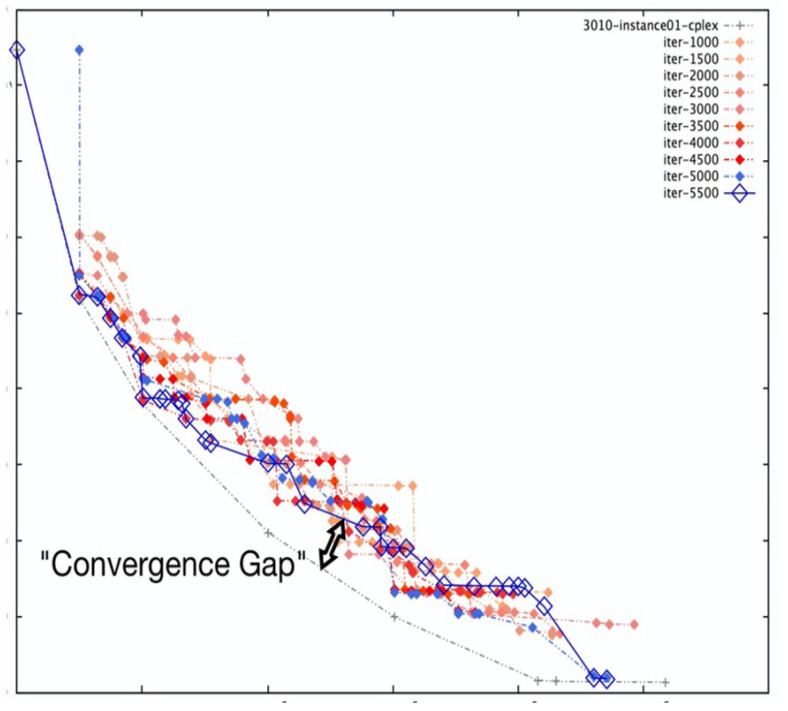
SMPSO algorithm struggles to converge into a true Pareto front.

On the other hand, SMPSO shows different characteristics. For the same problem set considered for NSGA-II, it can be observed from Figure 5 that SMPSO produces better results that comprehensively examine the entire front. This conserves the diversity in the solution set and produces Pareto fronts that are always evenly distributed. SMPSO, on the other hand, struggles to obtain the solution set converged into a true optimal front. However, for the same initial population size, SMPSO performs better than NSGA-II in terms of the coverage of Pareto fronts, but after a certain number of iterations, it still searches a different solution space instead of converging the solution towards the true optimal front. Whilst the solution quality can be substantially improved after an increased number of iterations, it results in a significant increase in computation time.

As a concluding remark, it can be said that NSGA-II achieves a better performance in terms of closeness to optimal solutions, while SMPSO performs better in terms of preserving the diversity in Pareto frontiers. However, none of these algorithms perform efficiently with regard to the diversity of solutions and the convergence gap towards the optimal front. Motivated by these drawbacks, in the next section, we propose a hybrid version of NSGA-II and SMPSO called a swarm-optimized non-dominated sorting Genetic Algorithm (SONG). The key objective of SONG is to exploit the searching efficiency of GA and PSO. In particular, the proposed SONG algorithm inherits the convergence and the diversity quality from NSGA-II and SMPSO, respectively.

## 6. Swarm Optimized Non-Dominated Sorting Genetic (SONG) Algorithm

The motivation of developing the SONG algorithm is to address the drawback of NSGA-II and SMPSO algorithms, which is also to combine the advantages of both these EMO algorithms. SONG is actually a hybrid version of these two algorithms wherein SMPSO precedes the NSGA phase in order to pre-process the Pareto optimal solution set. By embedding the NSGA operators in SMPSO, the balance between exploration and exploitation is significantly improved. To summarize, the SONG algorithm combines the ability of social thinking in the swarm algorithm with the local search capability of the NSGA-II algorithm. Because SMPSO and NSGA-II both are population-based algorithms, the SONG algorithm is also a population-based algorithm and is hence guaranteed to find the global solution.

The proposed SONG algorithm is given in Algorithm 3. In the initialization phase, the particles of the swarm and corresponding velocities are randomly generated over the search space. Thereafter, the position of each particle is updated according to Equation (27). After creating a new generation in the SMPSO phase, some particles of a new population are selected and NSGA-II is applied to each of them separately (line 18). Because the swarm size is often very large, it is not time efficient to employ NSGA over an entire population. Hence, out of the total swarm population, the number of swarm ($N_{nsga}$) that should evolve into a SMPSO renewal process is defined by:

(33) $$N_{nsga}=N_{nsga}^{max}-{\left(\frac{SMPSO_{i}}{SMPSO_{maxit}}\right)}^{\gamma }\cdot \left(N_{nsga}^{max}-N_{nsga}^{min}\right)$$

where $SMPSO_{i}$ and $SMPSO_{maxit}$ denote the maximum number of generations in SMPSO, respectively. After selecting the individual with the highest fitness (greedy selection), SONG creates a new population by replacing the individuals with improved fitness via selection, crossover, and mutation operators. Once a new population is evaluated, the population size $N_{nsga}^{pop}$ and maximum number of iterations $NSGA_{maxit}$ for the NSGA-II phase are updated with respect to each SMPSO iteration according to the following equations:

(34) $$N_{nsga}^{pop.}=N_{nsga}^{minpop}+{\left(\frac{SMPSO_{i}}{SMPSO_{maxit}}\right)}^{\gamma }\cdot \left(N_{nsga}^{maxpop}-N_{nsga}^{minpop}\right)$$

(35) $$NSGA_{maxit}=N_{nsga}^{minit}+{\left(\frac{SMPSO_{i}}{SMPSO_{maxit}}\right)}^{\beta }\cdot \left(N_{nsga}^{maxit}-N_{nsga}^{minit}\right)$$

**Algorithm 3** SONG Algorithm for facility location in VFC
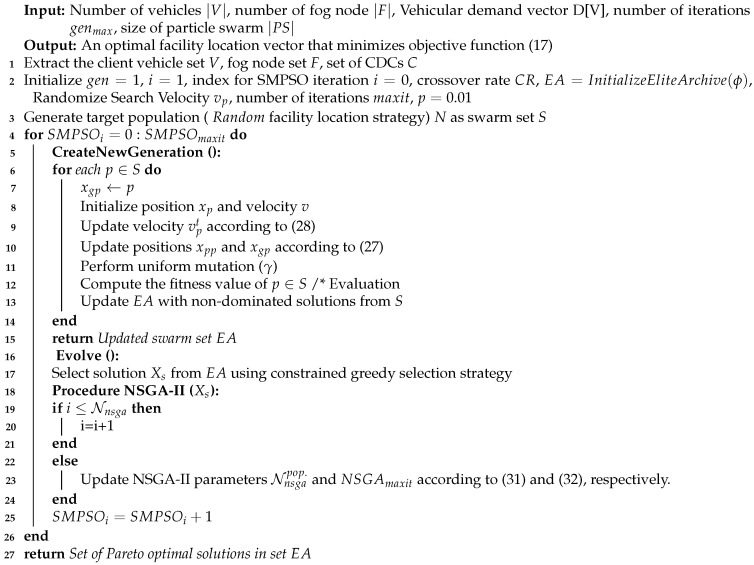


### Complexity Analysis

Denoting K = initial population size, *x* = mutation probability, N = number of objective functions, I = Max. Iterations. For each iteration, the SMPSO algorithm includes three key operations:

*Particle speed computation:*O(K)
*Uniform mutation operation:*O(x%·K)
*Polling for elite set:*$O(NK^{2})$

Thus, for I iterations, the complexity of the SMPSO procedure is $O(INK^{2})$. Similarly, for each iteration, the NSGA-II algorithm includes three operations:

*Non-dominated sorting:*$O(N{\left(2K\right)}^{2})$
*Crowding distance assignment:*O(N(2K)log(2K))
*Sorting and polling:*O(2Klog(2K))

Thus, for I iterations, the complexity of the NSGA-II procedure is $O(N{\left(2K\right)}^{2})$. Combining both phases, the overall complexity of the SONG algorithm becomes $O(INK^{2})$.

## 7. Evolutionary Algorithms Parametrization

### 7.1. Encoding Scheme for SONG

Generally, the EMO algorithm starts the reproduction process by generating an initial population pool. Each member in the initial population pool represents a potential solution to the optimization problem. In order to obtain a fitness value, these members are evaluated against a given objective and constraint function. Following this, different EMO algorithms apply different evolving processes to improve the fitness value of each member in the population. The whole process terminates when the predefined conditions are met. A solution will be selected as output only if it is not dominated by any members in the population (also known as non-dominated solutions) and do not violate any environment-specific (explicit) constraints.

While generating an initial population pool, the first step is to design a representative encoding scheme for the decision variables. The encoding scheme transfers multiple variables from a goal problem into a string of binary or integer values. The encoding technique has a significant effect on the EMO’s performance. The count-preserving encoding (CPE) [26] solves the problem arising in random encoding through a bit-count tracking scheme. In this work, we proposed a modified version of CPE (named as MCPE) where the same bit-count tracking scheme is employed but added with a novel facility capacity guarantee scheme. The facility capacity guarantee is enforced by applying the constraints (23) and (25) on each chromosome.

**Figure 6:**
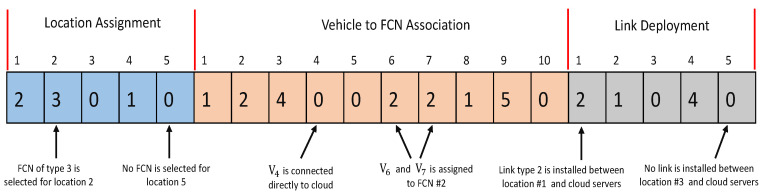
Proposed MCPE-based encoding string for *g*th chromosome.

For a VFC network with a |V| number of vehicles, the number of fog node |F| and |L| number of links, the dimension of chromosome (G) is (|V|+|F|+|L|). We assume that each fog node is connected to the remote CDCs through a single link of virtually infinite bandwidth, i.e., |F|=|L|. Hence, the overall dimension of chromosome (G) becomes (|V|+2|F|). In Figure 6, we illustrate an encoding string for the *g*th chromosome/particle in an FLP with 10 vehicles and 5 fog node locations. Suppose that $C_{g}$ is the *g*th particle of the population denoted by $C_{g}=\left[Z_{g,1},Z_{g,2}\ldots Z_{g,G}\right]$. Each component (gene) $Z_{g,h}\left(h\in G\right)$ of encoding string $C_{g}$ is defined as:

- For 1≤h≤|F|, $Z_{g,h}$ denotes whether an fog node is deployed at location *l*. Specifically, $Z_{g,h}=x$ indicates a fog node of type *x* is selected for location $l_{h}\in L$.
- For |F|+1≤h≤|F|+|V|, $Z_{g,h}$ denotes to which fog node type the workload from vehicle $v_{h}\in V$ is offloaded. Specifically, $Z_{g,h}=y$ indicates that $v_{h}$ is assigned to fog node *y*(y∈F), and $Z_{gh}=0$ indicates that $v_{h}$ is directly connected to the cloud.
- For |F|+|V|+1≤h≤2·|F|+|V|, $Z_{g,h}$ denotes the link decisions for the location *l*. Specifically, $Z_{g,h}=z$ indicates that a link of type *z* is installed at location *l*, whereas $Z_{g,h}=0$ indicates no link is placed for this location.

### 7.2. Operators

**Figure 7:**
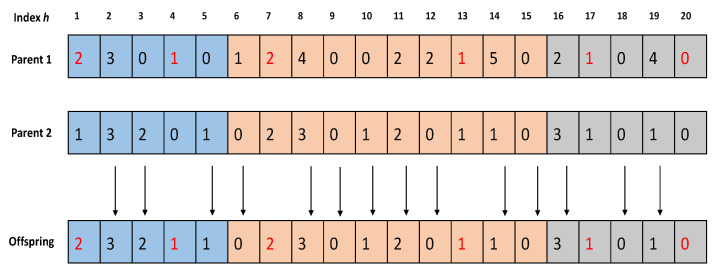
Knowledge-based crossover.

**Figure 8:**
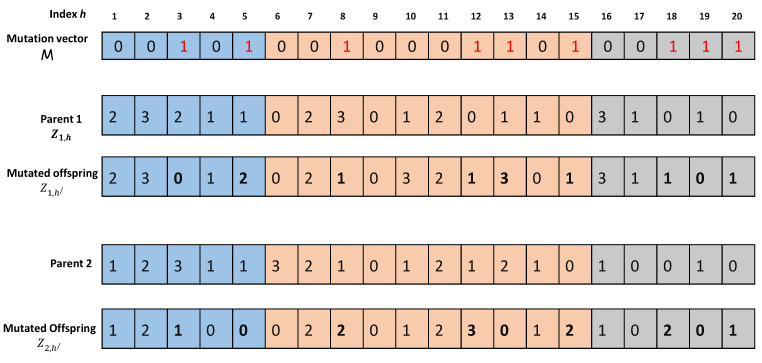
Uniform mutation.

- **Selection:** The SONG algorithm employs an aggressive selection process in the NSGA-II phase, which states that a child is selected for reproduction only if it is non-dominated or upgrades one fitness value achieved thus far. Because the SMPSO procedure preserves the population diversity, a harsh fitness process will not harm the solutions’ spread.
- **Crossover:** Generally, the population-based meta-heuristic algorithms employ a crossover operation for combining two parent chromosomes, with the hope of generating better-quality offspring. Unlike the standard crossover operator (viz. single point, binomial, and polynomial crossover), we define a knowledge-based crossover operator for NSGA-II phase in SONG. Here, the knowledge-based crossover operator selects two parents $\pi_{1}$ and $\pi_{2}$ according to an aggressive selection process described above in order to generate one offspring. To be specific, the local fitness of each gene is compared with the weighted combination of the average response time $\tau_{avg}$ and system energy consumption $e_{sys}$, given by Equation (17). The gene with better local fitness is copied to the offspring. The knowledge-based crossover operation is better depicted in Figure 7. Here, the genes marked in ’red’ represent smaller local fitness values compared with their parents. Hence, the genes at indexes 1, 4, 7, 13, 17, and 20 are copied from parent 1, and the rest genes are copied from parent 2.
- **Mutation:** The mutation operators modify chromosomes so as to improve the fitness values and avoid early convergence (i.e., to abstain from struck in local minima). It is a momentum variable which keeps the diversity of the population and avoids premature convergence. The SONG algorithm employs a uniform mutation where a binary mutation vector $M=(m_{1},m_{2}\ldots m_{L})$ is randomly generated. For each $m_{h}=1$, the value of gene $Z_{g,h}$ is flipped to a different value $Z_{g,h^{/}}$ where $h\neq h^{/}$. Figure 8 illustrates the uniform mutation for the mutation vector M=(0,0,1,0,1,0,0,1,0,0,0,1,1,0,1,0,0,1,1,1).

Each heuristic algorithm has several tunable hyperparameters, which can eventually have an impact on the quality of the final solution. Before running experiments on the generated datasets, the parameters of NSGA-II and SMPSO were tuned so that the best results can be obtained. For the NSGA-II algorithm, we compare the volume of the dominated space (HV) for four different parameters: the mutation distribution index, the crossover distribution index, mutation probability, and crossover probability. For tuning purposes, a population size of 100 according to [64] and 3000 iterations were used following the method in [29]. The basic idea is to test the number of iterations for EMO algorithms to reach 98% of the HV of the CPLEX’s Pareto front. We observed that all three EMOs can reach the 98% HV of CPLEX within 3000 iterations. Therefore, in parameter tuning tests, a population size of 100 with a number of iterations of 3000 is used for different settings.

Mutation probability is the probability to randomly change each decision variable. In our experiment, the value of MP takes a value in the range of [0,1] with a step size 0.1. Crossover probability (CP) is the probability of producing offspring from parent solutions. In our experiment, the value of CP takes value in the range of [0,1] with a step size of 0.1. We performed preliminary experiments on sample problems and observed that keeping a mutation probability as 0.1 shows the best performance. Similarly, setting the value of CP as 0.9 gives the best performance. We did not present the whole experiment since, as shown in Table 3, the value of 0.1 shows overall best performance.

The set of experiments similar to SMPSO parameter tuning were also performed to compare the volume of dominated space for two parameters: the mutation distribution index and mutation probability. The complete list of selected parameters for the NSGA II and SMPSO algorithm is presented in Table 3. The source codes for the NSGA-II, SMPSO, and SONG-based solutions are implemented in PYOMO (http://www.pyomo.org/, accessed on 21 November 2022), a Python-based open source package for solving multi-objective optimization problems. To perform the experiments, an Intel(R) Core (TM) workstation with i5-4590 CPU, 3.30 GHz internal clock and 8 GB RAM is used.

### 7.3. Settings for the Weighted Sum Method in ILOG CPLEX

We used the linearization technique to convert the problem P1 into a mixed integer linear programming (MILP) model. For creating baselines, we solved the model in an IBM ILOG CPLEX solver with default parameter settings (https://www.ibm.com/products/ilog-cplex-optimization-studio, accessed on 22 November 2022). For each problem instance, we generate 11 pair of weights with steps of size 0.1, the weight factors $\alpha_{1}$ and $\alpha_{2}$, where $\alpha_{1}+\alpha_{2}=1$ and the model is executed for 100 min. The weights factors $\alpha_{1}$ and $\alpha_{2}$, respectively, reflect the relative importance of an average response delay $\tau_{avg}$ and system energy consumption $e_{sys}$ on the overall optimization problem. For instance, in latency critical vehicular applications $\alpha_{1}$ will be relatively larger (or vice versa for a low power green computation models). The results obtained from the CPLEX solver are saved for to later be used as a reference for performance evaluation during EMO analysis.

## 8. Simulation Setup

In this section, we define the traffic scenario on which a case study is performed, followed by VANET configuration and other simulation parameterization for facility location in VFC, i.e., fog node characteristics, network topology, application characteristics and workload distribution, etc.

### 8.1. Traffic Simulation

**Figure 9:**
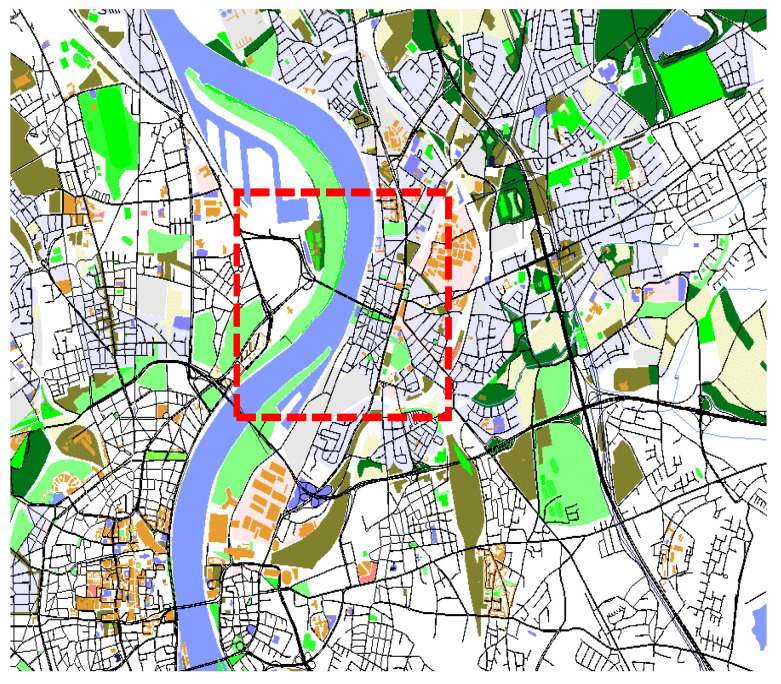
Map of Cologne considered for mobility generation. The bordered region contains 481 (rush hour) and 189 (idle hour) taxis and buses (VFN) and 67 RSUs.

In the proposed VFC architecture, each vehicle is considered to act either as a client node or a VFN. Hence, to simulate the mobility of mobile fog nodes, we used a set of real-world taxi and bus traces. Basically, we performed a case study of the data (traffic) management on a real-world city topology generated from the taxi and bus traces of Cologne [65]. Cologne is the third largest city of Germany by area and the fourth largest city by population density after Berlin, Hamburg and Munich. The mobility traces were created as part of the TAPASCologne project, an initiative of the ITS at the German Aerospace Center (ITS-DLR) [66]. The goal of this project is to simulate vehicular traffic in the greater urban area of Cologne, Germany, as realistically as possible. The dataset covers 4500 kilometers of roads in a 400-square-kilometer area and spans 24 h during a normal workday. It gives per-second information on the geographical coordinates and the speed of vehicles involved in 688,536 individual car trips. This is one of the most comprehensive mobility datasets to date that has been made freely available. The traces were obtained by integrating state-of-the-art tools devoted to VFC-specific attributes for traffic modeling in VANET. For VFC experiments, we retrieved the traces of only 1500 taxis/bus that include the longitude and latitude coordinates of GPS locations, heading directions, moving speeds, and record time-stamp. Because the vehicular distribution varies by zone, we consider one section of size 64 km^2^ from each of dense and sparse zone in Leverkusen (latitude 51.0603° N, longitude 6.97332° E). The output topology generated in SUMO (https://www.eclipse.org/sumo/, accessed on 21 November 2022) is shown in Figure 9, the where red encircled zone is considered for our study. In two time frames of 15 min (6.00–6.15 a.m. IST, 8.30–8.45 a.m. IST, 22 February 2022), 189 and 481 vehicles are observed in the rush hour and idle hour, respectively. To discover the locations of RSU, we considered the deployment of cellular BSs in Cologne, retrieved from public German databases in 2012 [65]. We used the same format for the RSU location information as we used for the vehicle positions, and is available at the same repository.

For solving the proposed facility location model using different EMO algorithms, we used a Python-based optimization package called PYOMO. We simulated the above-mentioned VANET scenario using Veins (https://veins.car2x.org/, accessed on 21 November 2022), an open source inter-vehicle communication (IVC) simulator. Basically, Veins connects the microscopic SUMO topology with an event-based network simulation framework called OMNET++ via Traffic Control Interface (TraCI) (https://sumo.dlr.de/docs/TraCI.html, accessed on 21 November 2022). DSRC and 5G communication are realized in SimuLTE (https://simulte.com/, accessed on 21 November 2022), an extension over the INET model library. It is an advanced simulation tool that enables a complex system-level performance evaluation of LTE and LTE advanced networks (3GPP Release 8 onwards). In our model, SimuLTE enables vehicles to either exchange data with vehicles or BS through IEEE 802.11p or IEEE 802.11bd, respectively. The overall simulation tools and the network configuration parameters are summarized in Table 4.

For modeling client vehicles, 150 vehicle routes are generated in SUMO using jtrouter (https://sumo.dlr.de/docs/jtrrouter.html, accessed on 21 November 2022), between times of 0 and 900 s. The vehicle flow density is maintained following Manhattan traffic model [67]. For each direction, the flow parameters are the maximum number of vehicles, the starting road and the destination of the flow, the time to start and end the flow and the probabilities of turning to different directions at each junction (0.3 to go straight, 0.25 to turn right and 0.25 to turn left). For each scenario, the workload (request/s) is generated by a constant bit rate (CBR) generator that lasts for 60 s. Each vehicle is assumed to be of same computing power. The processing power of fog nodes and vehicles are estimated to be 50 and 10 times more than that of PDs. Similarly, the cloud servers are assumed to be at least 200 times faster than that of FCN [32].

### 8.2. VANET System Configuration

For simulating inter-vehicular communication in the sparse zone, the IEEE 802.11p DSRC standard is used with a maximum data rate of 27 Mbps and approximately 300 m of coverage [12]. For dense vehicular zones, IEEE 802.11bd and 5G NR V2X are assumed to be the mode of communication among vehicles. The neighboring vehicles communicate with each other by broadcasting beacon messages through DSRC. However, vehicle-to-BS communication needs high mobility for the vehicles going at high speed. Thus, we use 5G NR V2X for high-speed vehicles moving at 350 km/h [8].

Based on the topology analysis in Section 8.1, we generated 25 problem instances to be used in EMO experimentation. The problem instances are generated by a program written in Python and are specified in Table 5. The task profile for each PD is specified according to Definition 1.

For the example problem Π(10,30,5,∞), we handle the uncertainty in location and velocity using fluid traffic model (FTM) [38] for modeling vehicular mobility. The FTM describes the output velocity $v_{s}$ of vehicle *v* as a monotonically decreasing function of vehicular density and enforces a lower bound on vehicle velocity when the traffic congestion reaches a critical state, guided by Equation (34).

(36) $$v_{s}=\min\left\{v_{s}^{min},v_{s}^{max}\left(1-\frac{k}{k_{jam}}\right)\right\}$$

where $v_{s}^{min}$ and $v_{s}^{max}$ are the minimum and maximum speed, respectively, $k_{jam}$ is the vehicular density for which a traffic jam is detected, and *k* is the current vehicular density of the road. We assume that the maximum speed $v_{s}^{max}$ of the vehicle is 40 km/h, and correspondingly, we randomly generate four instances for each problem size, over a location range (−100 and +100 m). This gives rise to a total of 100 problems. Each algorithm is then executed for four instances of each problem index/size. Finally, the values averaged over these four instances are presented. This goal here is to produce a better performance for each algorithm considering the randomness of each problem instance.

**Definition 5.**  *FLP Instance: In order to explain the working of a proposed FLP model, we defined an FLP instance by a quadruple Π(f,v,r,∞), where f indicates the number of fog locations, r represents the number of RSUs installed in that segment, v is the number of vehicles, and ∞ is for the remote cloud. For example, Π(6,12,4,∞) indicates that tasks from twelve vehicles need to be routed to six fog locations, in a road segment installed with fur RSUs.*

## 9. Results and Discussions

### 9.1. An Illustrative FLP Problem Π(10,30,5,∞)

**Figure 10:**
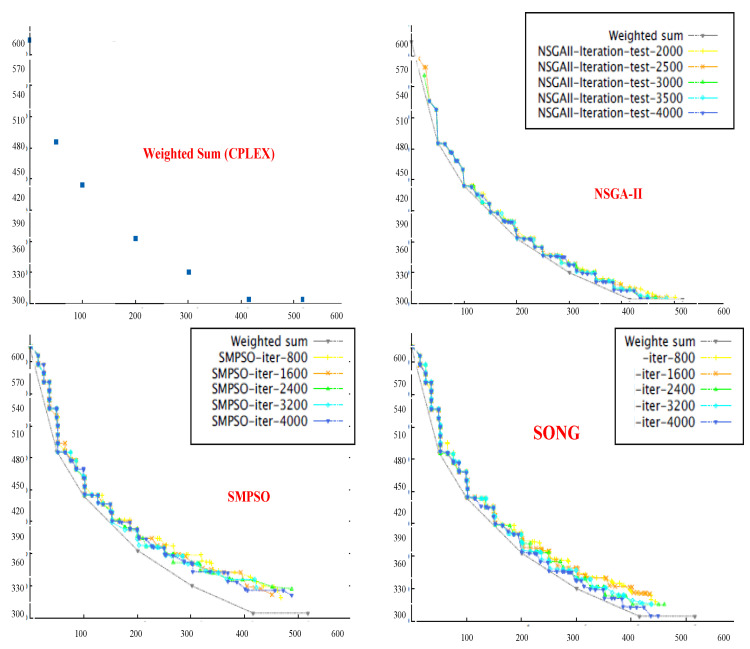
Solution frontiers for Π(10,30,5,∞).

To demonstrate the effectiveness of the proposed algorithm, we first evaluate the proposed SONG algorithm on an example problem Π(10,30,5,∞). We used this example to have a thorough analysis of the results to examine how a typical facility location model works for VFC. Here, one instance of Π(10,30,5,∞) is solved with the weighted sum method (CPLEX), NSGA-II, SMPSO and SONG algorithms. The nodes are assumed to be uniformly distributed in a (100×100 km^2^) area. The progress against the varying number of iterations and Pareto frontiers generated using CPLEX, NSGA-II, SMPSO, SONG, are plotted in Figure 10. It can be observed that, compared to NSGA-II which could not completely explore the solution space, the Pareto fronts in the SONG algorithm are uniformly distributed and are more extended along both axes. Similarly, compared to SMPSO where the solutions are far away from the optimal, the SONG solutions are sticking (nearest) to the optimal curve.

Each optimal solution indicates a different planning and routing scheme for the VFC network designers leading to trade-off between delay and energy.

For the problem Π(10,30,5,∞), it is also noteworthy that the multi-objective model gives several optimal solutions where different solution indices may converge to same objective function values. Each optimal solution indicates a different planning and routing scheme for the VFC network designers leading to a trade-off between delay and energy. For this example, we explained one solution where the optimal solution obtained from the CPLEX has a delay ($\tau_{avg}$) of 7.023 milliseconds and energy consumption ($e_{sys}$) value 2.32 unit. Basically, we plotted the topology layout generated for all four algorithms, corresponding to a fixed value of energy consumption of 77.31 units. In this case, we observed roughly identical solutions in each iteration as the search space is very small.

**Figure 11:**
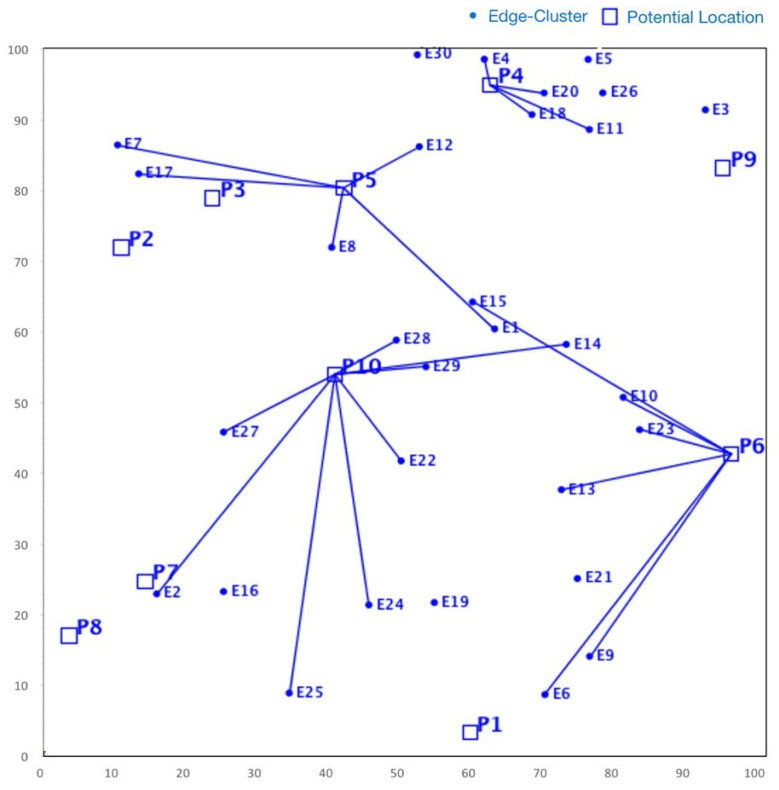
Output layout for CPLEX at $e_{v}^{avg}=77.31$ units, $\tau_{v}^{avg}$ 321.9 ms.

**Figure 12:**
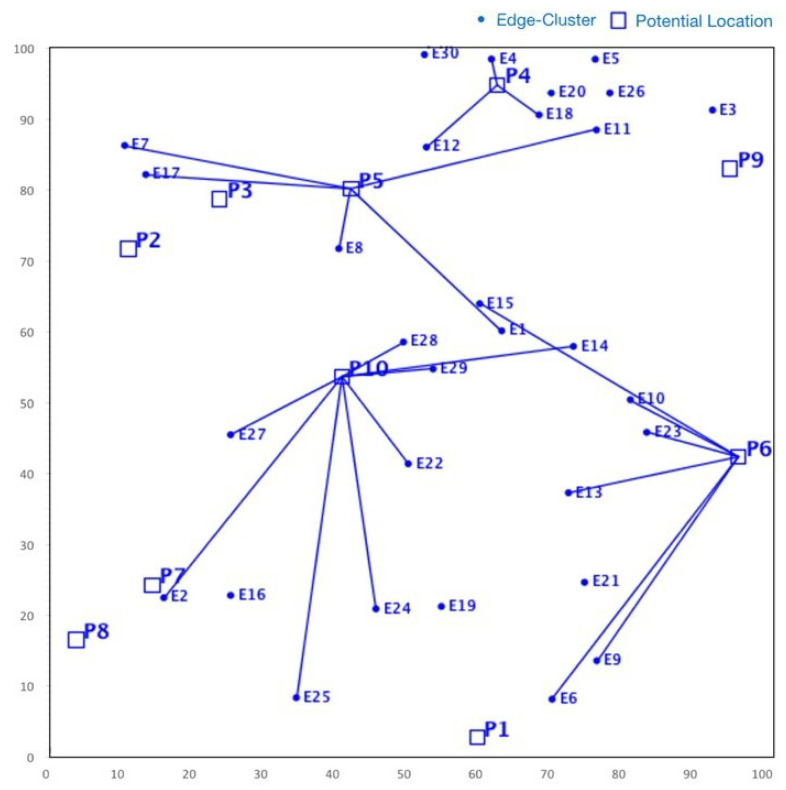
Output layout for NSGA-II at $e_{v}^{avg}=77.31$ units, 322.4 ms.

**Figure 13:**
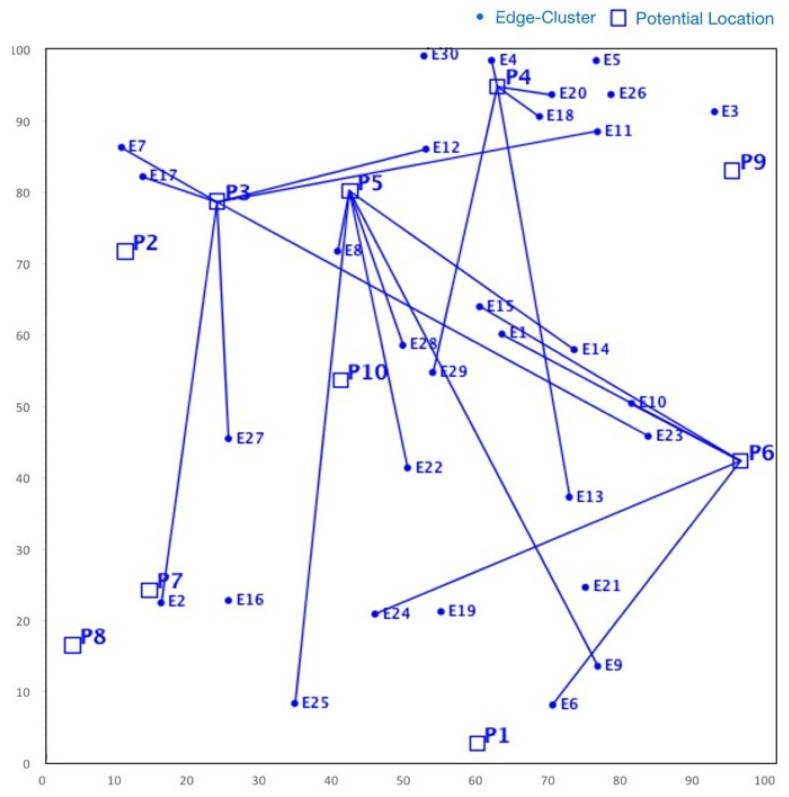
Output layout for SMPSO at $e_{v}^{avg}=77.31$ units, $\tau_{v}^{avg}$ 324.4 ms.

**Figure 14:**
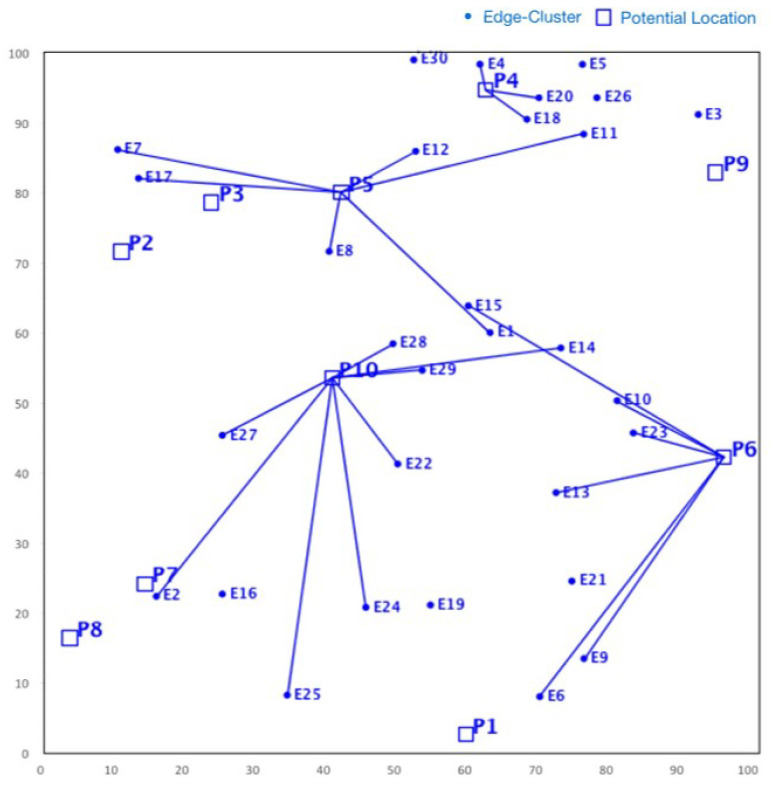
Output layout for SONG at $e_{v}^{avg}=77.31$ units, $\tau_{v}^{avg}$ 323.1 ms.

It can be observed from Figure 11, Figure 12, Figure 13 and Figure 14 that both NSGA-II and SONG generate nearly the same topologies as that of the weighted sum (CPLEX). For the same energy consumption (77.31 units), the delay for CPLEX, NSGA-II, SMPSO and SONG are 321.9 ms, 322.4 ms, 324.1 ms and 322.3 ms, respectively. The slight differences in delay are due to the variation in placement of a small number of vehicles (i.e., E11 and E12), and can be resolved through the efficient reallocation and load-balancing strategies.

Moreover, as the problem size increases, the solution time for CPLEX increases steeply. This is because CPLEX uses the branch and bound (B&B) algorithm which first splits the search space into smaller search spaces called *branching*. Then, it explores the sets of a smaller search space to obtain the minimum value of the objective function. The B&B strategy avoids the brute force search and testing by keeping track of bounds on the minimum value of the objective function. The bounds are then utilized to prune the search space by eliminating the candidate solutions that cannot give an optimal solution. In fact, the randomness in the nonlinear increase in the average solution time for a large problem size can also be related to the theory that the combinatorial solution points are pruned by the CPLEX solver during B&B heuristics. However, it is found that there is a wider gap for two different placement locations in the solution time. This is due to the NP-hardness of FLPs.

### 9.2. Performance Analysis on Real-World Transportation Datasets

An important issue in EMO is the qualitative analysis of the performance of different algorithms. There are numerous methods of measuring the quality of the solution for any optimization problem. The most common and simplest technique to measure the solution quality is to keep track of the objective functions and the time taken by the solver to reach an optimal solution. Generally, the time taken to reach an optimal solution must increase proportionally to the fleet size as the solver must consider a lot of combinations of where to install the fog nodes, what type of fog node needs to be installed, and which vehicle should be assigned to it. Usually, the outcome of an EMO algorithm is an approximation of the Pareto-optimal front, denoted as an approximation set. Hence, the question arises of how to evaluate the quality of those approximation sets. In this work, we performed a comparative analysis of SONG with the results obtained by a weighted sum method (CPLEX), NSGA-II and SMPSO in terms of three comparison metrics, namely hyper-volume (HV) indicator [33], delay gap, and inverted generational distance (IGD) [29]. For performance evaluation, we solved the facility location model for 25 different problems defined in Table 5.

EMO algorithms tend to create a set of non-dominated solutions to form Pareto fronts, which introduces uncertainty into decision makers’ preference. To deal with the uncertainty, a compromised solution on the Pareto fronts of each problem using fuzzy systems is used [68], which models the fuzziness in the goal of each objective function. The contribution of Pareto fronts to each objective is modeled as a fuzzy membership function which has values in the range of [0, 1]. The best compromising solution is found when the solutions in a Pareto front are very close to each other. The membership value for the *j*th solution for the *i*th objective in the Pareto front is calculated using the membership function:

(37) $$\mu_{i}^{j}=\left\{\begin{array}{c} 1\;if\;F_{i}\leq F_{i}^{min}\\ \frac{F_{i}^{max}-F_{i}}{F_{i}^{max}-F_{i}^{min}}\;if\;F_{i}^{min}<F_{i}<F_{i}^{max}\\ 0\;F_{i}\geq F_{i}^{max} \end{array}\right.$$

where $\mu_{i}^{j}$ indicates how well the *j*th solution is able to satisfy the *i*th objective in a Pareto optimal set. The sum of all the membership values for all objectives of the *j*th solution indicates how well it satisfies all the objectives. Given Υ solutions in a Pareto front and χ objective functions for each solution, the achievement of each solution with respect to all the Υ solutions can be calculated by:

(38) $$\mu^{j}=\frac{\sum_{i=1}^{\chi }\mu_{i}^{j}}{\sum_{j=1}^{\Upsilon }\sum_{i=1}^{\chi }\mu_{i}^{j}}$$

The best compromise solution accepted by the decision maker is the solution which has a maximum value of $\mu^{j}$.

#### 9.2.1. Comparison of Hyper-Volume (HV) Indicator

The volume enclosed by a Pareto approximation and a reference set is called the HV indicator, which is also called Nadir point. The reference point usually represents the worst possible point in the solution set. It explores both the convergence and diversity properties of a solution set. Search strategies in EMO are usually evaluated using reference points because it helps reduce complexity in density-based EMO and also follows strict monotonicity in Pareto dominance [22]. *A set with a large HV is always desirable as it presents a better set of trade-offs*.

**Figure 15:**
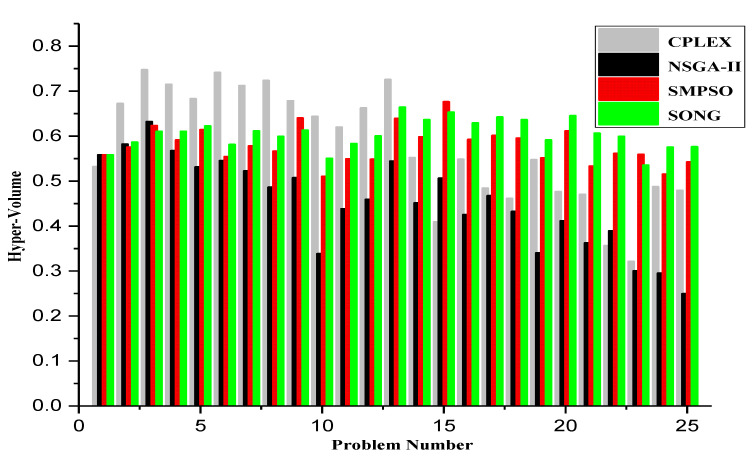
Comparison of HV indicators.

From Figure 15, it can be observed that the SONG algorithm achieves superior results over other algorithms for the majority of problem sizes. We noticed that among the 100 solved problems, SONG gives the highest HV value for 89 problems (89%). The performance of the weighted sum from CPLEX is poor due to the fact that a branch and bound search can only generate a single solution. It is also to be noted that the performance of NSGA-II decreases as the problem size increases. This is due to two reasons. First, the search space is large in large-scale problems. Second, the non-dominated sorting in the NSGA-II algorithm has a concentrated effect. Basically, the SONG algorithm exhibits improved results because of the cooperative optimization between the SMPSO phase and the NSGA-II phase. It exploits the advantages of both SMPSO as well as NSGA-II, i.e., the former explores the decision space and preserves the diversity in the solution frontiers whereas the latter gives emphasis to the convergence of the solution pool towards the optimal Pareto fronts.

#### 9.2.2. Comparison for IGD Indicators

The notion of generational distance (GD) was introduced in [69] as a metric to estimate how far the elements obtained in the Pareto front are from those in the true Pareto front of the problem. The IGD is an “inverted” version of GD that utilizes the true Pareto front as a reference set and compares each of its elements with respect to the front produced by an algorithm. For a non-dominated vector $X=\{x_{1},x_{2},\ldots x_{\left|X\right|}\}$ and reference vector $Y=\{y_{1},y_{2},\ldots y_{\left|Y\right|}\}$, the IGD is defined as:

(39) $$IGD\left(X\right)=\frac{1}{\left|Y\right|}\sqrt{\left(\sum_{i=1}^{\left|Y\right|}d_{i}^{2}\right)}$$

where $d_{i}$ is the Euclidean distance (measured in objective space) between each member of the solution set $x_{i}$ and the nearest member of the reference set *Y*. Since the IGD is a measure of distance, *the lower the value of IGD, the better the quality of solution set is*. In our analysis, the best non-dominated solutions returned by all three methods (NSGA-II, SMPSO, SONG {P=50}) are used as a reference set.

In Table 6, we provide the values of the IGD quality indicator obtained for the weighted sum (CPLEX), NSGA-II, SMPSO and SONG algorithms. It can be seen that the SONG algorithm gives better solution quality (lower IGD values) over other three algorithms for more than 90% of problems. Such an improvement over NSGA-II and SMPSO can be defined by the information exchange between the PSO and GA phases in the SONG algorithm. Smaller values of IGD values shown by the SONG algorithm for the majority of FLP problem instances proves that the Pareto fronts obtained are better than the other three algorithms.

#### 9.2.3. Delay Gap Comparison

**Figure 16:**
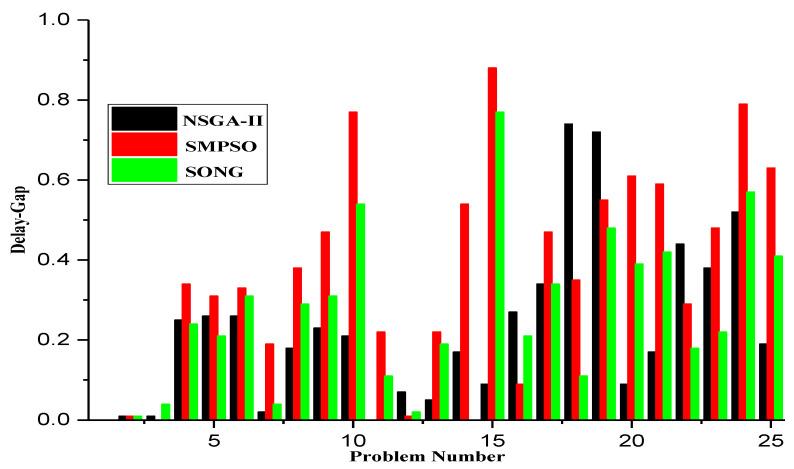
Delay Gap Comparison with reference to CPLEX.

As discussed in Section 7.3, for each problem, with a scale of 0.1, we generate 11 different weight combinations for the WS method. Correspondingly, we obtain the decision output for the link type and the fog type at each location. We also calculate the relative mixed-integer programming (MIP) gap, i.e., the percentage gap between the solution frontier obtained by CPLEX and the value of the optimum for each problem instance. Without loss of generality, it can be concluded that all solutions provided by the CPLEX solver are optimal solutions. In other words, given a fixed value of energy consumption, the delay produced by the weighted sum and CPLEX can obtain the optimum solution. Now, considering CPLEX solutions as the reference, we compute the average delay gaps between CPLEX vs. NSGA II, SMPSO and SONG. It is noteworthy that the delay gaps were calculated only if the weighted sum solution frontiers has the same fitness and CPLEX obtained the optimal solutions, i.e., the relative MIP is equal to 0. It can be observed from Figure 16 that NSGA-II provides the minimal delay gap to optimal solutions because of the non-dominated sorting procedure that concentrates the solution sets on the optimal front. Interestingly, the delay gap for SONG is the second minimum (best) for the majority of problems.

## 10. Conclusions

In this paper, we studied the FLP in VFC networks. Specifically, we provided a multi-objective formulation of the FLP to minimize the service delay and system energy consumption. To solve this problem, we developed a hybrid EMO algorithm called SONG, which combines the convergence speed of NSGA-II, and the diversity efficiency of SMPSO algorithm. We show that, although the SONG algorithm theoretically has the same complexity as NSGA-II and SMPSO, for a facility location in VFC, it gives improved results. Furthermore, to evaluate its empirical performance, we computed the values of three quality indicators, namely: HV, IGD, and delay gap, for each of these algorithms. We execute the SONG algorithm on an example problem as well as on real-world mobility datasets and observed that SONG exhibits a better solution quality than the NSGA-II and SMPSO algorithm. In particular, the SONG-based facility location framework will enable the VFC service providers as well as transportation planners to evaluate the pros and cons of each of the trade-off solutions, leading to informed network planning and design decisions. Moreover, because we consider the mobility of fog nodes through topology generation from real-world vehicular traces, the proposed framework will provide more flexibility, especially when there is uncertainty in the traffic demand.

The peculiar property of VFC to integrate the Internet of Vehicles (IoT), sensor fusion, and V2X communication is attributed to its wide application in the transportation industry. We believed that the analytical studies conducted in this work will pave better ways for researchers and ITS planners to develop VFC services for future connected and autonomous vehicle (CAV) driving beyond 5G networks. However, the current formulation is an offline model, i.e., it assumes all the workloads are known in advance. Although such a model can be extremely useful for benchmarking, it cannot be used in a real-time environment because some of the information, e.g., the start time, end time, task duration, etc., are not available at the time to make the decision. One possible approach to address such an issue is to develop online heuristics that dynamically assign tasks to fog nodes whenever they arrive, and simultaneously manage the status of all fog nodes. We keep this as a scope of our future study.

## Figures and Tables

**Table 1:** Summary of the literature in facility location or service placement problem in VFC or fog architectures.

Ref.	Objective Function	Decision Outputs	Algorithm	Mobility Support
[8]	Delay	Task offloading	Binary PSO	Yes
[37]	Delay	Service placement	ILP	No
[38]	Delay minimization	Task offloading	SMDP/Bellman equation	Yes
[39]	Delay	Service placement	ILP	No
[40]	Delay	Service placement	Petri nets	No
[43]	Delay	Task scheduling	NSGA-II	No
[44]	Delay	Task scheduling	Tabu Search	No
[45]	Delay	Task offloading	Simulated annealing	No
[46]	Cost	Service placement	ILP	No
[47]	Cost	Service placement	Heuristics	No
[48]	Cost	Service migration	MDP	No
[49]	Cost	Service placement	MDP	No
[6]	Cost	Service placement	MILP	Yes
[51]	Energy consumption	Service placement	Monte Carlo	No
[52]	Energy consumption	Service placement	ILP	No
[53]	Energy consumption	Task scheduling	Fuzzy reinforcement learning	No
[20]	Delay and energy consumption	Task offloading	Hungarian algorithm	No
[54]	Delay and energy consumption	Task offloading	Genetic algorithm	No
[55]	Delay and energy consumption	Task offloading	Heuristics	No
[56]	Delay and energy consumption	Task scheduling	Deep neural network	No
[12]	Delay and energy consumption	Task offloading	Differential evolution	Yes
Our work	Delay and energy consumption	Service placement	SONG	Yes

**Table 2:** Acronyms and symbols used in this paper.

**Acronym**	**Description**
PDs	Passenger devices
BSs	Base stations
HV	Hyper-volume
VFC	Vehicular fog computing
RSUs	Roadside units
VFN	Vehicular fog node
IGD	Inverted generational distance
IoT	Internet of Things
SCN	Storage, computing and networking
IDC	International Data Corporation
FLP	Facility location problem
QoS	Quality of service
ITSs	Intelligent transportation systems
CAVs	Connected and autonomous vehicles
EMO	Evolutionary multi-objective optimization
CAGR	Compound annual growth rate
NSGA	Non-Dominated Sorting Genetic Algorithm
VANET	Vehicular ad hoc network
SMPSO	Speed-constrained particle swarm optimization
**Symbol**	**Description**
*v*, *V*	Index for set of vehicles
*f*, *F*	Index for set of vehicles fog nodes
*c*, *C*	Index for set of vehicles cloud servers
$r_{v}^{a}$	Workload for vehicle *v* (bytes/s.)
$r_{f}^{a}$	Traffic arrival rate for fog node *f*
$r_{c}^{a}$	Traffic arrival rate for cloud server *c*
$r_{f}^{s}$	Service rate of fog node *f*
$r_{c}^{s}$	Service rate of cloud server *c*
$\sigma_{v}$	Task size (bytes) *v*
$E_{f}$	Energy consumption per CPU cycle of fog node *f*
$C_{f}$	Computing capacity of fog node *f*
${ℜ}_{v}$	Up-link data rate for vehicle *v*
$P_{p}^{tran}$	Transmission power of PD *p*
$POF_{v}$	Processor occupancy factor of vehicle *v*

**Table 3:** Parameter selection for different phases of the SONG algorithm.

Parameters	NSGA II	SMPSO
$C_{1}$, $C_{2}$	-	1.5, 1.5
Inertia (*w*)	-	
$N_{nsga}^{max}$, $N_{nsga}^{min}$	20, 1	-
γ, β	10, 15	-
$N_{nsga}^{minpop}$, $N_{nsga}^{minit}$	10, 10	-
Mutation distribution index	18	14
Mutation probability	0.02	-
Crossover distribution index	18	-
Crossover probability	0.9	-

**Table 4:** Network specification and simulation parameters.

Parameters	Specifications
$\Pi_{v}$	0.1
$POF_{v}$	0.3
$r_{p}^{a}$	U[1, 2] MIPS
$r_{v}^{cap}$	5 MIPS
|F|	481 (dense), 189 (sparse)
|V|	150
Speed of VFN	[2, 5] km/h
Client Vehicle speed	[20, 40] km/h
$\sigma_{v}$	U[32, 128] bytes
$\tau_{v}^{max}$	50–700 ms
$C_{f}$ (MIPS)	50 (cars) & 100 (bus)
$\omega_{v,f}$	20 MHz (802.11p, WAVE)
	410 MHz (802.11bd, 5G NR (FR1))
$r_{c}^{s}$	$10^{5}$ MIPS
$P_{p}^{tran}$	23 dBm
$\alpha_{v}$	2605 mJ/s
$A_{c}$	{3.2, 4.4, 2.3}
$B_{c}$	{68, 53, 70}
*u*	U[2.5, 3]
$n_{c}$	U[200, 500]
OMNET++ Simulation time	60 s

**Table 5:** Problem instances for SONG experiments.

Problem No.	# Fog Nodes	# Vehicles	# RSU	Cloud
1	5	10	1	*∞*
2	5	15	1	*∞*
3	5	20	2	*∞*
4	5	25	2	*∞*
5	10	30	2	*∞*
6	10	40	2	*∞*
7	10	50	5	*∞*
8	10	60	5	*∞*
9	10	70	5	*∞*
10	10	80	5	*∞*
11	15	70	8	*∞*
12	15	80	8	*∞*
13	15	90	8	*∞*
14	15	100	8	*∞*
15	15	100	10	*∞*
16	15	100	10	*∞*
17	15	120	10	*∞*
18	15	140	10	*∞*
19	15	160	10	*∞*
20	15	180	10	*∞*
21	20	200	10	*∞*
22	20	250	10	*∞*
23	20	300	10	*∞*
24	20	350	10	*∞*
25	20	400	10	*∞*

**Table 6:** Comparison of IGD values.

Prob. No.	CPLEX	NSGA-II	SMPSO	SONG
1	0.294	0.0201	0.0024	0.0013
2	0.147	0.00922	0.0026	0.0012
3	0.0958	0.00594	0.0019	0.00096
4	0.0372	0.0124	0.0026	0.00146
5	0.0411	0.013	0.0021	0.00291
6	0.03572	0.0135	0.0035	0.00315
7	0.0743	0.0121	0.0053	0.00613
8	0.0992	0.224	0.00498	0.00448
9	0.0843	0.0168	0.00259	0.00229
10	0.112	0.0173	0.00554	0.00454
11	0.134	0.0208	0.00444	0.00344
12	0.0391	0.0157	0.00532	0.00502
13	0.0521	0.00178	0.00355	0.00325
14	0.0705	0.0176	0.0024	0.00194
15	0.0427	0.0151	0.00237	0.00167
16	0.124	0.017	0.00311	0.002311
17	0.112	0.0152	0.00353	0.003153
18	0.179	0.0176	0.00351	0.00321
19	0.181	0.0164	0.00343	0.003043
20	0.297	0.0192	0.00386	0.00426
21	0.353	0.0153	0.00259	0.00289
22	0.303	0.0179	0.00334	0.00304
23	0.438	0.0197	0.00355	0.002955
24	0.403	0.0156	0.00459	0.003459
25	0.683	0.0198	0.00641	0.00681

## Data Availability

The datasets used for the research work can be provided on request.
